# The Roles of RNA Polymerase I and III Subunits Polr1c and Polr1d in Craniofacial Development and in Zebrafish Models of Treacher Collins Syndrome

**DOI:** 10.1371/journal.pgen.1006187

**Published:** 2016-07-22

**Authors:** Kristin E. Noack Watt, Annita Achilleos, Cynthia L. Neben, Amy E. Merrill, Paul A. Trainor

**Affiliations:** 1 Stowers Institute for Medical Research, Kansas City, Missouri, United States of America; 2 Department of Anatomy and Cell Biology, University of Kansas Medical Center, Kansas City, Kansas, United States of America; 3 Center for Craniofacial Molecular Biology, Ostrow School of Dentistry, University of Southern California, Los Angeles, California, United States of America; 4 Department of Biochemistry and Molecular Biology, Keck School of Medicine, University of Southern California, Los Angeles, California, United States of America; Albert Einstein College of Medicine, Schneider Children's Hospital, UNITED STATES

## Abstract

Ribosome biogenesis is a global process required for growth and proliferation of all cells, yet perturbation of ribosome biogenesis during human development often leads to tissue-specific defects termed ribosomopathies. Transcription of the ribosomal RNAs (rRNAs) by RNA polymerases (Pol) I and III, is considered a rate limiting step of ribosome biogenesis and mutations in the genes coding for RNA Pol I and III subunits, *POLR1C* and *POLR1D* cause Treacher Collins syndrome, a rare congenital craniofacial disorder. Our understanding of the functions of individual RNA polymerase subunits, however, remains poor. We discovered that *polr1c* and *polr1d* are dynamically expressed during zebrafish embryonic development, particularly in craniofacial tissues. Consistent with this pattern of activity, *polr1c* and *polr1d* homozygous mutant zebrafish exhibit cartilage hypoplasia and cranioskeletal anomalies characteristic of humans with Treacher Collins syndrome. Mechanistically, we discovered that *polr1c* and *polr1d* loss-of-function results in deficient ribosome biogenesis, Tp53-dependent neuroepithelial cell death and a deficiency of migrating neural crest cells, which are the primary progenitors of the craniofacial skeleton. More importantly, we show that genetic inhibition of *tp53* can suppress neuroepithelial cell death and ameliorate the skeletal anomalies in *polr1c* and *polr1d* mutants, providing a potential avenue to prevent the pathogenesis of Treacher Collins syndrome. Our work therefore has uncovered tissue-specific roles for *polr1c* and *polr1d* in rRNA transcription, ribosome biogenesis, and neural crest and craniofacial development during embryogenesis. Furthermore, we have established *polr1c* and *polr1d* mutant zebrafish as models of Treacher Collins syndrome together with a unifying mechanism underlying its pathogenesis and possible prevention.

## Author Summary

Ribosomes synthesize all proteins, and are therefore critical for cell growth and proliferation. Ribosome biogenesis, or the process of making ribosomes, is one of the most energy consuming processes within a cell, and disruptions in ribosome biogenesis can lead to congenital disorders termed ribosomopathies. Interestingly, individual ribosomopathies are characterized by tissue-specific phenotypes, which is surprising given the universal importance of ribosomes. Treacher Collins syndrome (TCS) for example, is a ribosomopathy characterized by anomalies of facial bones, palate, eyes and ears. Mutations in *TCOF1*, *POLR1C*, and *POLR1D* are associated with the underlying etiology of TCS. *TCOF1* plays an important role in the synthesis of ribosomal RNA, one of the rate-limiting steps of ribosome biogenesis. Consequently, *TCOF1* is essential for the survival and proliferation of neural crest cell progenitors, which are the precursors of craniofacial bone, cartilage and connective tissue. In contrast, the functions of POLR1C and POLR1D, which are subunits of RNA Polymerases I and III remain unknown. Here we examined the function of *polr1c* and *polr1d* during zebrafish development and discovered that these genes display dynamic spatiotemporal activity during embryogenesis with enriched expression in craniofacial tissues. Furthermore, we observed that *polr1c* and *polr1d* loss-of-function zebrafish exhibit anomalies in craniofacial cartilage development, which reflects the characteristic features of TCS. An examination of *polr1c*^-/-^ and *polr1d*^-/-^ mutants revealed that diminished ribosome biogenesis results in neuroepithelial cell death and a deficiency of migrating neural crest cells, which are the progenitors of the craniofacial skeleton. Moreover, the cell death observed in *polr1c*^-/-^ and *polr1d*^-/-^ mutants is Tp53-dependent, and inhibition of *tp53* is sufficient to repress cell death and rescue cranioskeletal cartilage formation in *polr1c*^-/-^ and *polr1d*^-/-^ mutant embryos. These studies provide evidence for tissue-specific functions of *polr1c and polr1d* during embryonic development, while also establishing *polr1c* and *polr1d* loss-of-function zebrafish mutants as models of Treacher Collins syndrome.

## Introduction

Ribosomes are large ribonucleoprotein complexes that translate mRNA, thus synthesizing all the proteins within a cell. The process of making ribosomes, which is known as ribosome biogenesis, takes place within the nucleolus and begins with the transcription of ribosomal RNAs (rRNAs) by RNA Polymerases I and III (RNA Pol I and III). RNA Pol I transcribes the 47S precursor rRNA which is subsequently processed into 18S, 5.8S, and 28S rRNAs, while RNA Pol III transcribes the 5S rRNA [1]. Transcription of the 47S rRNA is one of the rate-limiting steps of ribosome biogenesis, and accounts for about 60% of all cellular transcription in eukaryotes [2]. Ribosome biogenesis is a complex and metabolically expensive endeavor that universally governs the quality and quantity of all cellular proteins in all cells, and is therefore highly regulated by, and integrated with, cell growth, proliferation and differentiation [3,4].

Disruptions in ribosome biogenesis often result in disorders of embryonic development or adult homeostasis, which are collectively termed ribosomopathies [5]. Given the ribosome’s universal importance in all cells, it is surprising that ribosomopathies exhibit very specific clinical phenotypes which may include defects in the craniofacial, axial, and/or limb skeleton as well as in hematopoiesis or organogenesis. In addition, considerable variability exists within the phenotypic spectrum of individual ribosomopathies, which presents a considerable challenge to our understanding of the etiology and mechanistic pathogenesis of these conditions.

Treacher Collins syndrome (TCS, MIM 154500; TCS2, MIM 613717; TCS3, MIM 248390) is a rare congenital disorder of craniofacial development. TCS is characterized by hypoplasia of the facial bones, particularly the mandible and zygomatic complex, together with cleft palate, downward slanting of the palpebral fissures, and anomalies of the external and middle ear. Interestingly, there is a considerable degree of phenotypic variability in the severity and combination of these characteristic anomalies both between and within families [6,7]. TCS occurs with an estimated incidence of 1:50000 live births and is primarily associated with autosomal dominant mutations in *TCOF1* [8]. *TCOF1* encodes a putative nucleolar phosphoprotein termed treacle, which functions in the initiation of transcription by RNA Pol I as well as in rRNA processing [9,10]. Mice with heterozygous mutations in *Tcof1* phenocopy the cranioskeletal anomalies observed in humans with TCS including retrognathia, micrognathia and cleft palate [11]. Furthermore, *Tcof1* has been shown to play a critical role in the survival and proliferation of neuroepithelial and neural crest progenitor cells, which generate most of the craniofacial skeleton [11,12]. Collectively these results imply that ribosome biogenesis may be dynamically or spatiotemporally regulated and furthermore that neural crest cell progenitors exhibit a specific threshold sensitivity to deficiencies in ribosome biogenesis.

Recently, mutations in *POLR1C* and *POLR1D* were also found to underlie the etiology of TCS [13]. The mutations in *POLR1C* were autosomal recessive, while mutations in *POLR1D* were either autosomal dominant or autosomal recessive [13,14]. *POLR1C* and *POLR1D* encode subunits common to RNA Pol I and RNA Pol III, which transcribe rRNAs [2], however the precise functional roles for POLR1C and POLR1D in ribosome biogenesis and embryonic development, as well as in the pathogenesis of TCS, remain to be determined. In order to understand the roles of POLR1C and POLR1D, we characterized the spatiotemporal activity of *polr1c* and *polr1d* during zebrafish embryogenesis and investigated the phenotype of *polr1c* and *polr1d* homozygous mutant zebrafish with a particular emphasis on craniofacial development. We discovered that *polr1c* and *polr1d* are spatiotemporally and dynamically expressed, particularly during craniofacial development, and consistent with this pattern of activity, *polr1c* and *polr1d* homozygous mutant zebrafish exhibit cartilage hypoplasia and cranioskeletal anomalies characteristic of TCS. Mechanistically, we discovered that *polr1c* and *polr1d* loss-of-function perturbs ribosome biogenesis, resulting in Tp53-dependent neuroepithelial cell death and a deficiency of migrating neural crest cells, which underpins the cranioskeletal defects. More importantly, we show that genetic inhibition of *tp53* can suppress neuroepithelial cell death and ameliorate the cranioskeletal anomalies in *polr1c*^-/-^ and *polr1d*^-/-^ mutants, providing a potential avenue to prevent the pathogenesis of TCS. Our work has therefore revealed tissue specific roles for *polr1c* and *polr1d* during embryogenesis and more specifically in craniofacial development. Furthermore, we have established *polr1c*^-/-^ and *polr1d*^-/-^ mutant zebrafish as models of TCS, while also unifying the underlying biochemical and cellular disease mechanisms as well as avenues for possible prevention.

## Results

### *polr1c* and *polr1d* are dynamically expressed during craniofacial development

**Fig 1:**
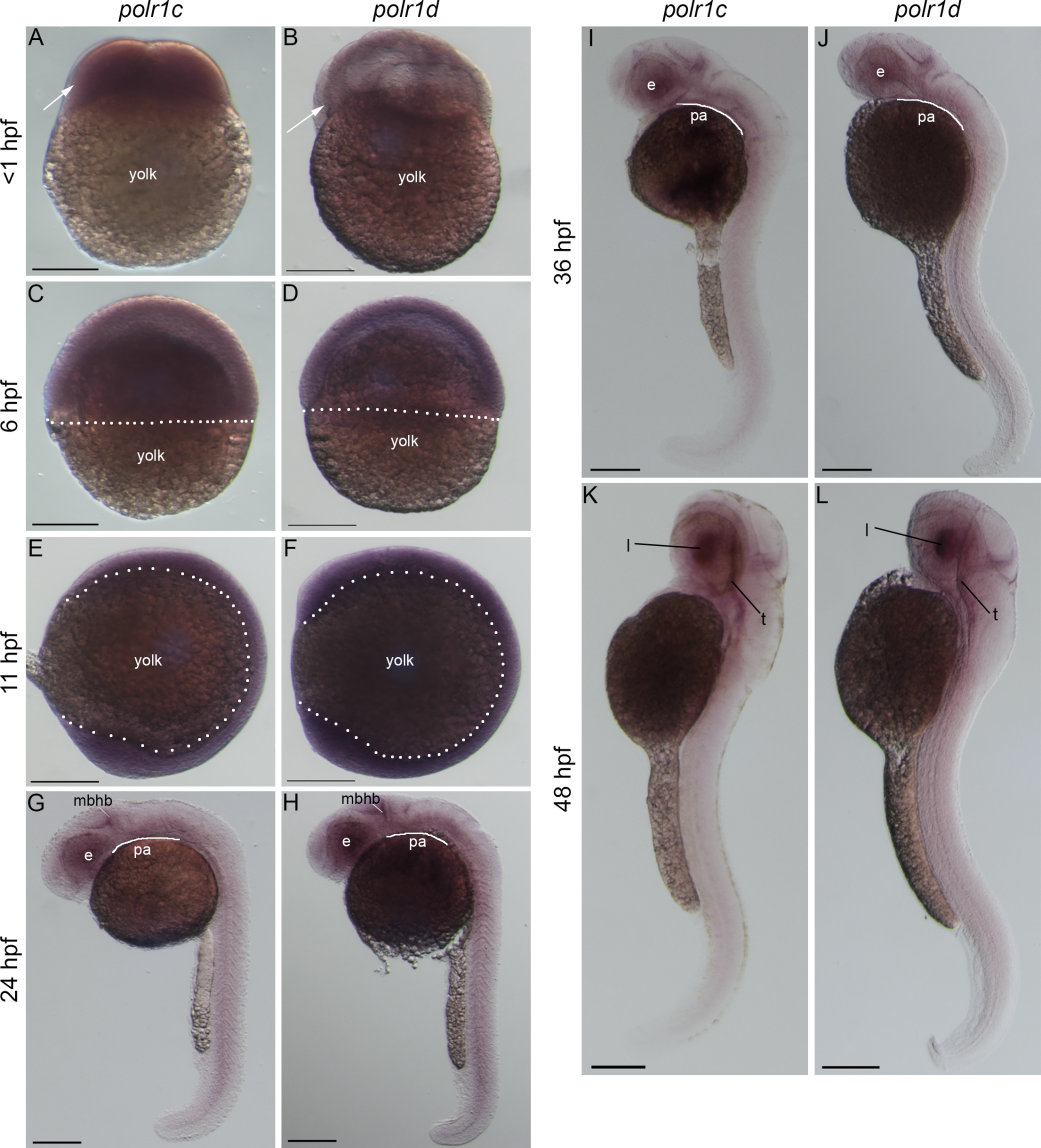
*polr1c* and *polr1d* are dynamically expressed during zebrafish embryogenesis. *polr1c* and *polr1d* are maternally expressed at early stages (A,B, arrows) and ubiquitously expressed at 6 hpf (C,D) and 11 hpf (E,F) when the embryo surrounds the yolk (dashed lines). At 24 hpf, expression becomes enriched in regions such as the eye and midbrain-hindbrain boundary (G,H). Elevated levels of expression are evident in the pharyngeal arches (adjacent to curved line) at 36 hpf (I,J) whereas lower levels are observed throughout the embryo at 48 hpf and beyond (K,L) and beyond. Abbreviations: e, eye; mbhb, midbrain-hindbrain boundary; pa, pharyngeal arches; l, lens; t, tectum. Scale bar = 200 μm.

To begin to understand the roles of *polr1c* and *polr1d* in craniofacial development, we characterized the activity of these genes during zebrafish embryogenesis (Fig 1). *polr1c* and *polr1d* are maternally expressed at stages <1 hour post fertilization (hpf) and remain ubiquitously expressed through gastrulation (6 hpf) and early neurulation (11 hpf) (Fig 1A–1F). A more dynamic pattern of *polr1c* and *polr1d* expression emerges by 24 hpf, with enriched domains of activity in the eye, midbrain, and central nervous system (Fig 1G and 1H). In 36 hpf zebrafish embryos, elevated levels of expression persist within the eye and discrete regions of the brain. In addition, enriched expression is evident in the pharyngeal arches, which will eventually give rise to the craniofacial cartilages (Fig 1I and 1J). At 48 hpf, *polr1c* and *polr1d* continue to be expressed at very low levels broadly throughout the embryo, however high levels remain in the lens and tectum (Fig 1K and 1L). These analyses collectively demonstrate that RNA Pol I and III subunits such as *polr1c* and *polr1d*, exhibit surprisingly dynamic spatiotemporal patterns of activity during embryogenesis. This suggests there may be tissue-specific threshold requirements for rRNA transcription during development and furthermore that *polr1c* and *polr1d* may play functional roles in craniofacial morphogenesis.

### Mutations in zebrafish *polr1c* and *polr1d* result in craniofacial anomalies

**Fig 2:**
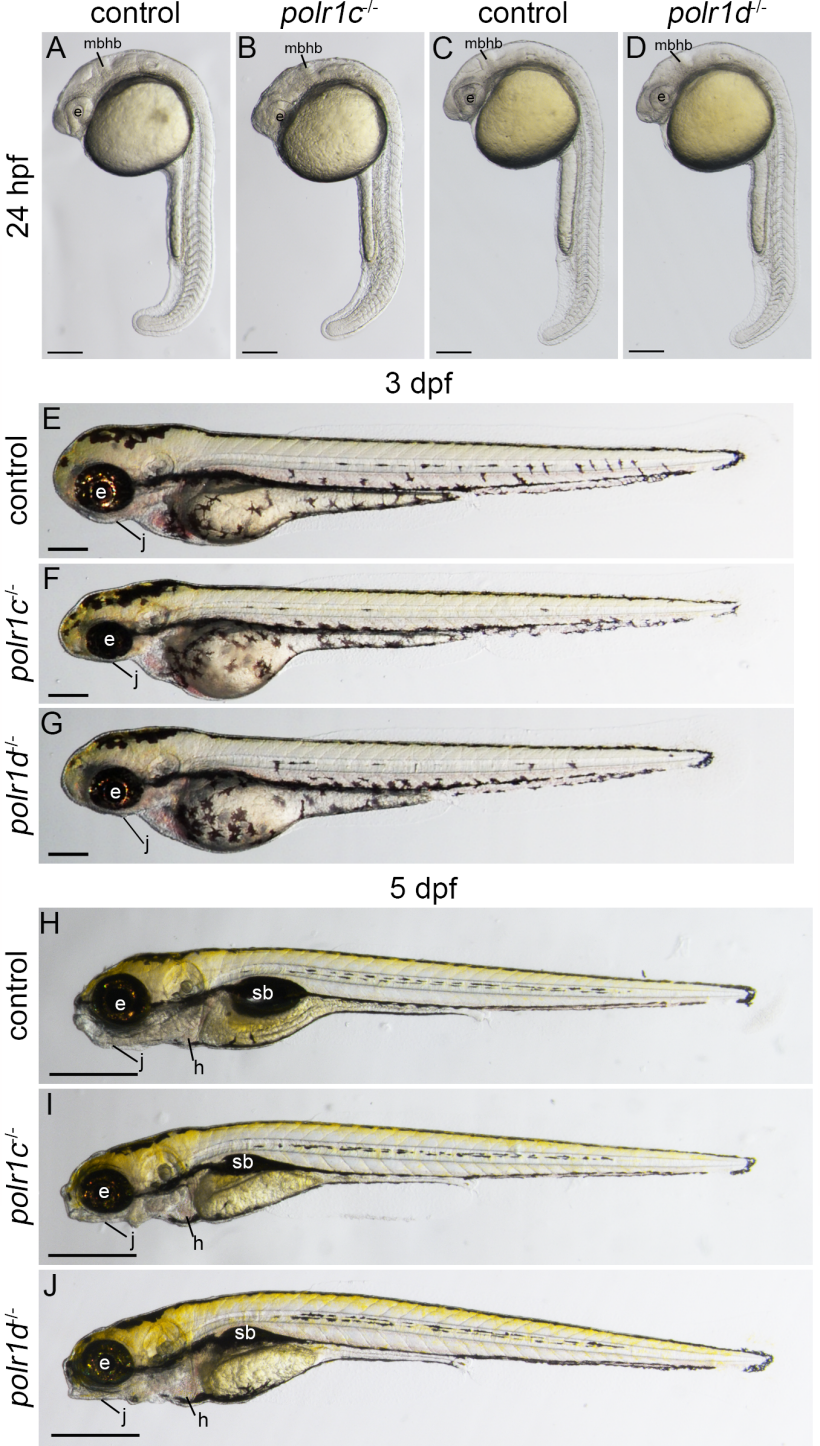
Mutations in *polr1c* and *polr1d* disrupt craniofacial development in zebrafish embryos. 24 hpf (A-D), 3 dpf (E-G) and 5 dpf (H-J) *polr1c*^-/-^ and *polr1d*^-/-^ zebrafish exhibit craniofacial defects, including smaller eyes, a disrupted midbrain-hindbrain boundary and cranial necrosis compared to controls. At 3 dpf, distinct craniofacial anomalies such as a smaller jaw and eyes become apparent. By 5 dpf, *polr1c*^-/-^ and *polr1d*^-/-^ mutants are distinguished from their control siblings by their smaller head, microphthalmia, jaw hypoplasia, and failure to inflate their swim bladder (E-G). Abbreviations: e, eye; mbhb, midbrain hindbrain boundary; j, jaw; h, heart; sb, swim bladder. Scale bar = 200 μm (A-G) and 500 μm (H-J).

To test our hypothesis that RNA polymerase subunits exert tissue-specific roles during embryogenesis, we characterized the phenotype of two mutant zebrafish lines: *polr1c*^hi1124Tg^ and *polr1d*^hi2393Tg^ hereafter referred to as *polr1c*^-/-^ and *polr1d*^-/-^ respectively. These zebrafish lines were generated by insertion mutagenesis which disrupts the transcription of each gene [15]. The mutation in *polr1c* lies in exon 2 while the mutation in *polr1d* is located in the first intron (http://web.mit.edu/hopkins/). These mutations dramatically reduce the levels of *polr1c* and *polr1d* transcripts during embryogenesis (S1 Fig). Homozygous *polr1c*^-/-^ and *polr1d*^-/-^ mutant embryos are phenotypically distinguishable from their control siblings at least as early as 24 hpf by their smaller eyes, disrupted midbrain-hindbrain boundary and necrotic cranial tissue (Fig 2A–2D). Interestingly, these affected structures are consistent with the tissue domains where *polr1c* and *polr1d* are primarily expressed, supporting an autonomous role for RNA Pol I. At 3 dpf, *polr1c*^-/-^ and *polr1d*^-/-^ mutants are distinguishable from control siblings by their smaller heads, microphthalmia, and hyploplastic jaws (Fig 2E–2G). By 5 days post fertilization (dpf), the craniofacial anomalies in *polr1c*^-/-^ and *polr1d*^-/-^ mutants become more pronounced (Fig 2H–2J). Although overall body size is comparable between mutant embryos and control siblings, *polr1c*^-/-^ and *polr1d*^-/-^ mutants present with a considerably smaller head together with mandibular hypoplasia and microphthalmia. *polr1c*^-/-^ and *polr1d*^-/-^ mutant embryos develop pericardial edema, and fail to inflate their swim bladders. Subsequently, both *polr1c*^-/-^ and *polr1d*^-/-^ mutant embryos die between 9–10 dpf.

**Fig 3:**
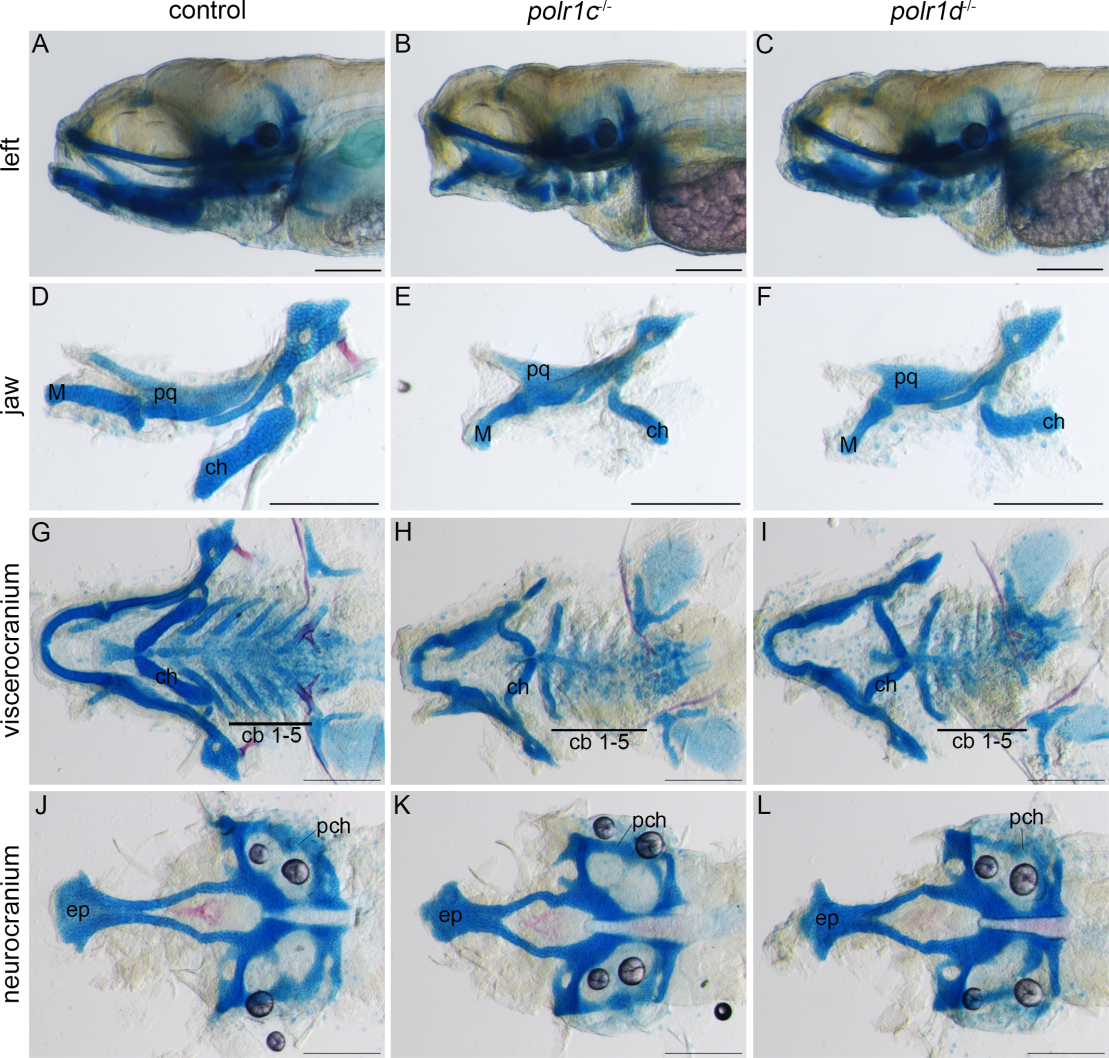
Craniofacial cartilage development is disrupted in *polr1c*^-/-^ and *polr1d*^-/-^ mutant embryos. (A-C) Alcian blue staining reveals cranial cartilage in 5 dpf *polr1c*^-/-^ and *polr1d*^-/-^ mutant embryos is hypoplastic compared to controls. (D-F) The jaws of mutant embryos are smaller overall, with noticeable differences in the size of Meckel’s cartilage, the palatoquadrate, and ceratohyal elements. (G-I) Staining of the viscerocranium reveals smaller cartilage elements derived from each of the pharyngeal arches in mutant embryos, most notably the ceratobranchials, as well as altered polarity of the ceratohyal. (J-L) Staining of the neurocranium reveals hypoplasia of the ethmoid plate. Abbreviations: M, Meckel’s cartilage; pq, palatoquadrate; ch, ceratohyal; cb, ceratobranchial; ep, ethmoid plate; pch, parachordal. Scale bar = 200 μm.

To further characterize the extent of craniofacial defects in *polr1c*^-/-^ and *polr1d*^-/-^ mutant embryos, we stained their cartilage with Alcian blue. In 5 dpf mutant embryos, the craniofacial cartilages are severely hypoplastic (Fig 3A–3C). Consistent with the morphology of a smaller jaw, hypoplasia of individual cartilage elements such as the palatoquadrate and Meckel’s cartilage was also observed (Fig 3D–3F), mimicking characteristic features of TCS in humans. The ceratohyal was similarly hypoplastic and exhibited reversed polarity in *polr1c*^-/-^ and *polr1d*^-/-^ mutants. Furthermore, the posterior pharyngeal arch derived ceratobranchials that comprise part of the viscerocranium exhibit very little Alcian blue staining, which is further evidence for cartilage hypoplasia (Fig 3G–3I). In the neurocranium, the ethmoid plate is smaller in mutant embryos compared to controls, however the parachordal cartilages appear to be of normal size. (Fig 3J–3L). By 9 dpf, all the craniofacial cartilage elements in *polr1c* and *polr1d* mutant embryos appear hypoplastic compared to controls (S2 Fig). Collectively, these craniofacial anomalies mimic the primary characteristic features of TCS in humans. This establishes *polr1c*^-/-^ and *polr1d*^-/-^ mutant zebrafish as potential models for understanding the pathogenesis of TCS, while also providing evidence for tissue-specific roles of RNA polymerase I and III subunits during embryogenesis.

### NCC development is disrupted in *polr1c*^-/-^ and *polr1d*^-/-^ mutant embryos

**Fig 4:**
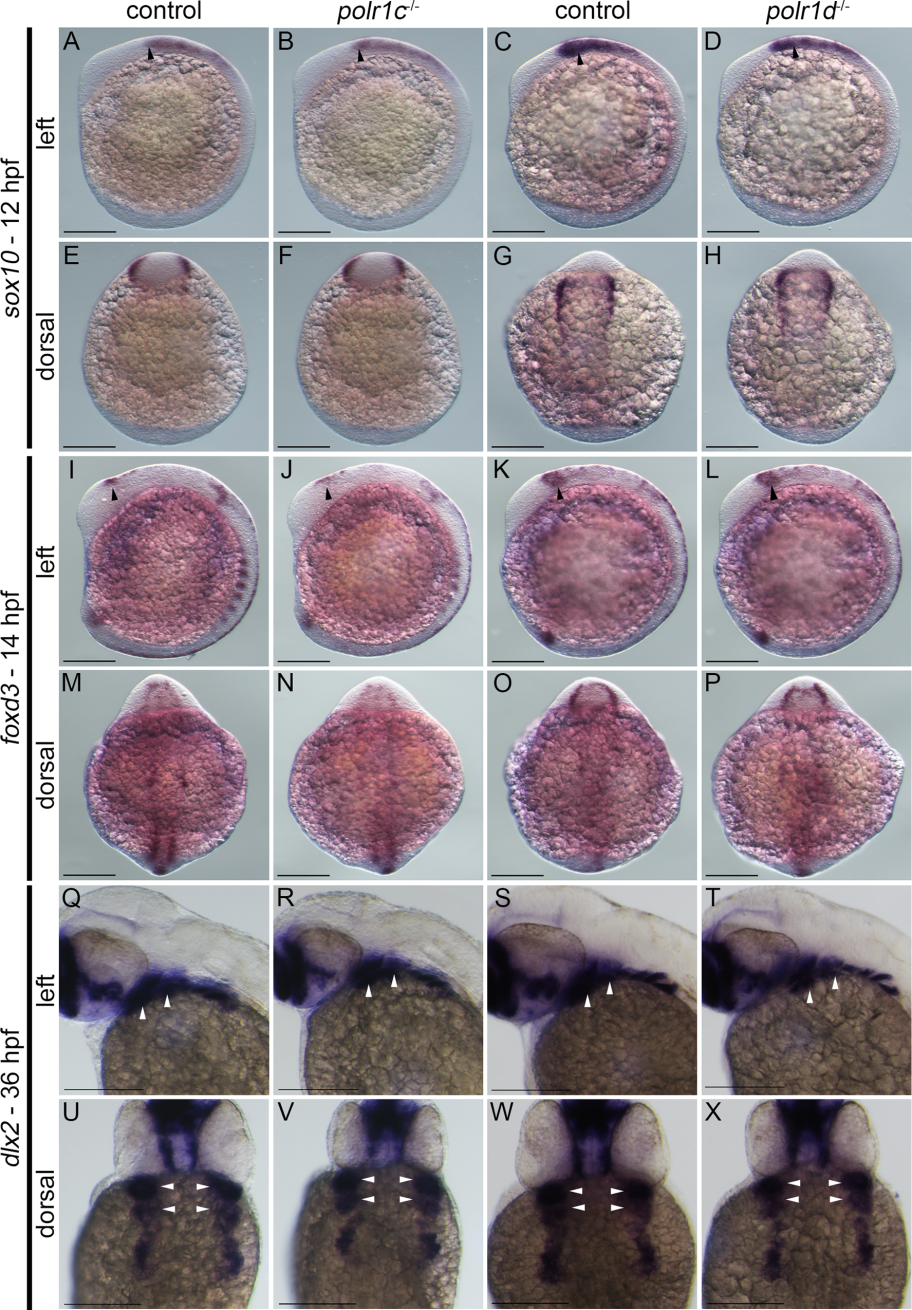
Analysis of NCC development in *polr1c* and *polr1d* mutant embryos. (A-H) *sox10* expression at 12 hpf and (I-P) *foxd3* expression at 14 hpf reveal relatively normal patterns of early cranial NCC specification and migration in *polr1c*^-/-^ and *polr1d*^-/-^ embryos (black arrows). (Q-X) In contrast, *dlx2* expression at 36 hpf reveals slightly diminished domains of activity in mutant embryos, particularly with respect to the posterior pharyngeal arches, which is suggestive of fewer mature NCC colonizing the pharyngeal arches. White arrows indicate pharyngeal arches 1 and 2. Scale bar = 200 μm.

The craniofacial skeleton in zebrafish is derived from both neural crest cells (NCC) and mesoderm [16,17]. Our observations indicate that NCC-derived structures of the viscerocranium and neurocranium are malformed in *polr1c*^-/-^ and *polr1d*^-/-^ mutants. In contrast the parachordal cartilages, which are of mesoderm origin were unaffected. We therefore hypothesized that *polr1c* and *polr1d* loss-of-function may specifically affect NCC development and thus underpin the cellular pathogenesis of craniofacial anomalies in *polr1c*^-/-^ and *polr1d*^-/-^ mutant zebrafish. To test our hypothesis, we initially investigated whether the neural plate, the progenitor tissue from which NCC are derived, was specified properly in *polr1c*^-/-^ and *polr1d*^-/-^ mutant embryos. Using *sox2* as a marker of definitive neural plate formation and specification, we observed similar *sox2* expression and patterning of the neural plate in 11hpf *polr1c*^-/-^ and *polr1d*^-/-^ mutant embryos compared to controls (S3 Fig). This suggests that *polr1c* and *polr1d* are not necessary for neural plate formation. To identify anomalies in early NCC development, we examined premigratory and migratory NCC through in situ hybridization with *sox10* and *foxd3* respectively, which are genes known to play important roles in NCC formation, survival, migration, and fate determination [18,19]. The spatiotemporal patterns of *sox10* (Fig 4A–4H) and *foxd3* activity (Fig 4I–4P) in premigratory and migratory NCC were very similar in *polr1c*^-/-^ and *polr1d*^-/-^ mutants compared to control siblings. However, using *dlx2* as a marker of mature cranial NCC as they colonize the pharyngeal arches and complete their migration [20], we observed smaller domains of expression particularly with respect to the caudal-most pharyngeal arches (Fig 4Q–4X). Although the expression levels of *sox10*, *foxd3*, and *dlx2* appeared to be normal in *polr1c*^-/-^ and *polr1d*^-/-^ mutants, indicating that the specification and migration of NCC occurred properly, we hypothesized that smaller territories of *dlx2* expression were indicative of reduced numbers of migrating NCC colonizing the pharyngeal arches. Furthermore, we posited that reduced numbers of migrating NCC could account for the cranioskeletal hypoplasia observed in 5 dpf *polr1c*^-/-^ and *polr1d*^-/-^ mutants (Fig 3).

**Fig 5:**
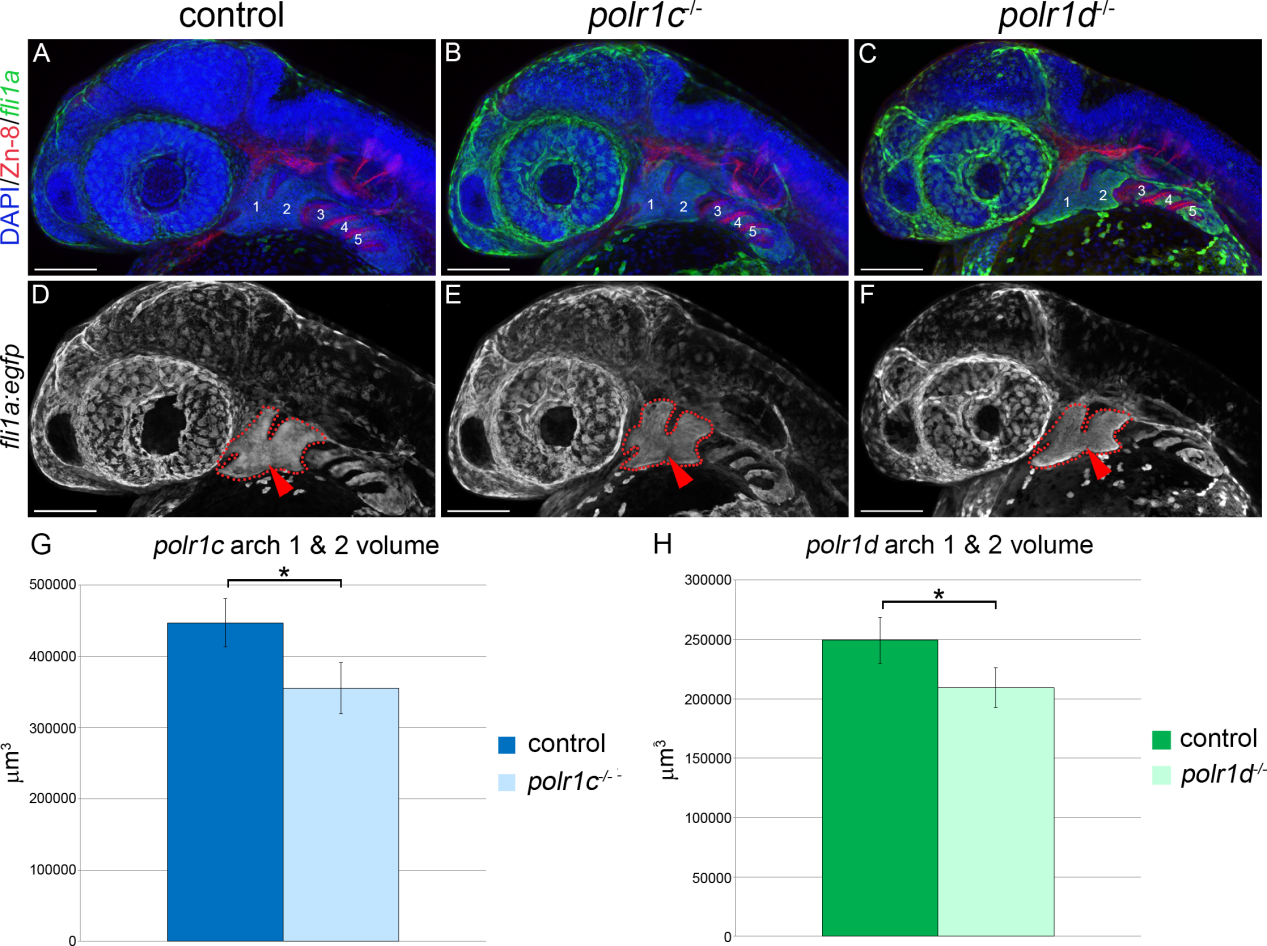
Pharyngeal arch size is reduced in *polr1c*^-/-^ and *polr1d*^-/-^ mutants. (A-C) Immunostaining of 36hpf *fli1a*:*egfp* labeled control, *polr1c*^-/-^ and *polr1d*^-/-^ mutant embryos with Zn-8 (red), which labels the endodermal pouches, revealed comparatively normal pharyngeal arch and pharyngeal pouch patterning. Pharyngeal arches 1–5 are indicated. (D-F) *fli1a*:*egfp* labeling of post-migratory NCC illustrates an overall reduction in pharyngeal arch size in *polr1c*^-/-^ and *polr1d*^-/-^ mutants. Pharyngeal arches 1 and 2 are outlined in red. (G-H) Quantification of pharyngeal arches 1 & 2 (red arrows, D-F) volume revealed a reduction in *polr1c*^-/-^ (G) and *polr1d*^-/-^ (H) mutants. Scale bar = 100 μm. * = p < 0.05 and error bars represent 95% confidence intervals.

To further validate our hypothesis that a deficiency in migrating NCC and pharyngeal arch hypoplasia underpins the cranioskeletal malformations in *polr1c*^-/-^ and *polr1d*^-/-^ mutants, we investigated the structure and composition of the pharyngeal arches. Endodermal pouches are known to play an important role in cranioskeletal patterning and differentiation [21]. To rule out the possibility that a defect in endodermal pouch patterning was responsible for the phenotype in *polr1c*^-/-^ and *polr1d*^-/-^ mutant embryos, we bred *fli1a*:*egfp*, which labels post-migratory NCC that colonize the branchial arches, into the background of *polr1c*^-/-^ and *polr1d*^-/-^ mutant zebrafish and immunostained with Zn-8, which marks the endodermal pouches [22,23]. We observed no alteration in the formation or segregation of the endodermal pouches in 36 hpf *polr1c*^-/-^ and *polr1d*^-/-^ mutants as evidenced by a normal pattern of Zn-8 activity (Fig 5A–5C).

In contrast, *fli1a*:*egfp* labeling of post-migratory NCC in combination with volumetric rendering revealed a significant reduction in the size of the pharyngeal arches in *polr1c*^-/-^ and *polr1d*^-/-^ mutant embryos (Fig 5D–5F). *polr1c*^-/-^ embryos exhibited an average volume of 3.55 x 10^5^ μm^3^ in contrast to 4.47 x10^5^ μm^3^ in control siblings (p = 0.0088, t-test; Fig 5G). Similarly, *polr1d*^-/-^ embryos exhibited a volume of 2.09 x 10^5^ μm^3^ in contrast to 2.49 x 10^5^ μm^3^ in control siblings (p = 0.022, t-test; Fig 5H). Thus the volume of pharyngeal arches 1 and 2 in *polr1c*^-/-^ and *polr1d*^-/-^ mutant embryos was reduced by approximately 20% compared to controls. Moreover, this is consistent with the apparently smaller domains of *dlx2* expression, which was also indicative of reduced pharyngeal arch size (Fig 4Q–4X). Since the specification and migration of NCC appears to occur normally in *polr1c*^-/-^ and *polr1d*^-/-^ mutant embryos as evidenced by *sox10* and *foxd3* expression, this implies that pharyngeal arch hypoplasia is the result of an overall reduction in the number of NCC colonizing the pharyngeal arches. Consequently, we hypothesized that increased apoptosis and/or decreased proliferation might account for these reduced cell and tissue populations.

### Cell death is increased in *polr1c* and *polr1d* mutant embryos

**Fig 6:**
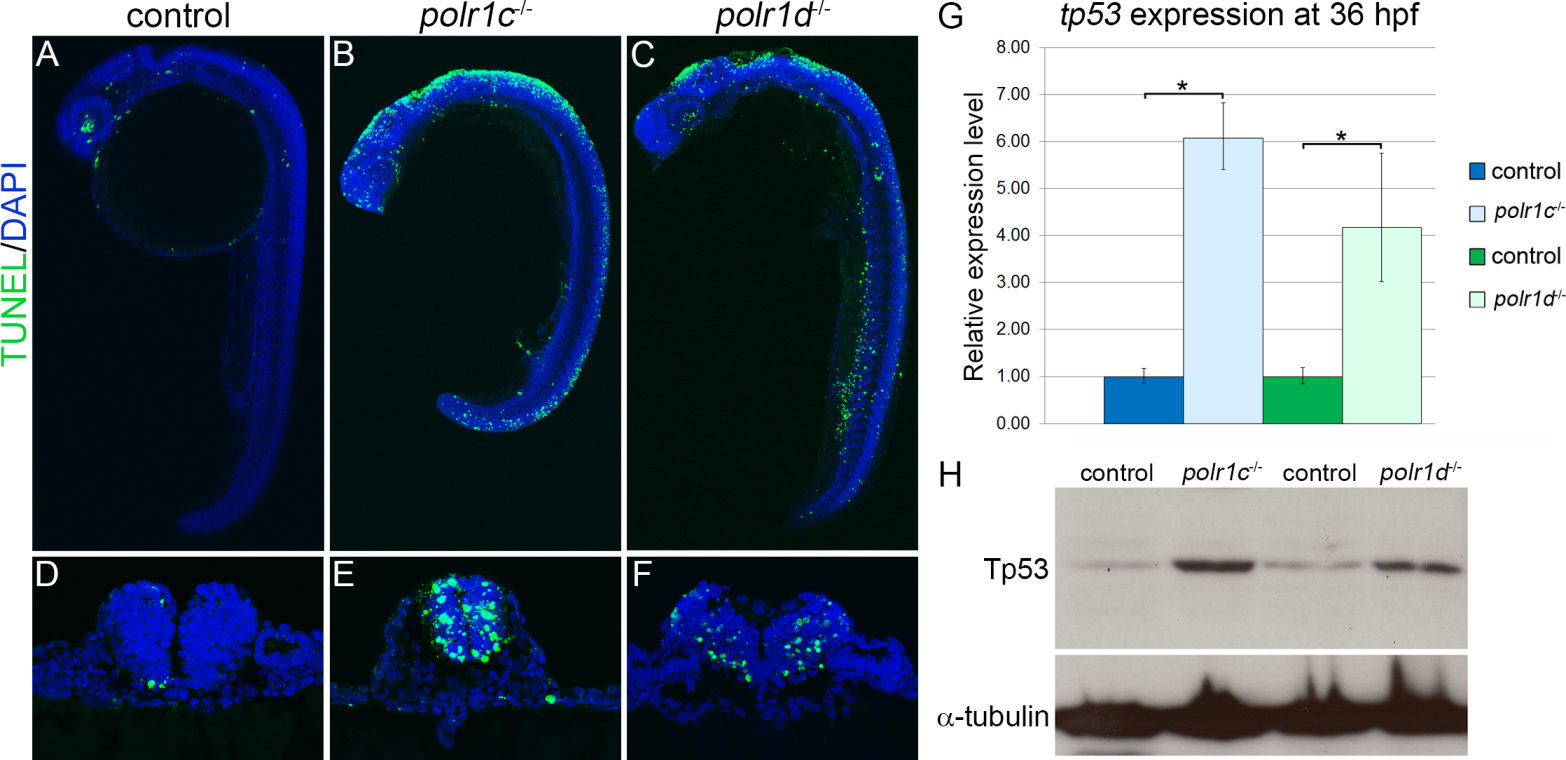
Increased Tp53-dependent cell death within the neuroepithelium of *polr1c*^-/-^ and *polr1d*^-/-^ embryos. (A-C) TUNEL staining (green fluorescence) revealed increased cell death in the cranial region and dorsal tissues of 24 hpf *polr1c*^-/-^ and *polr1d*^-/-^ mutant embryos. (D-F) Transverse sections revealed that cell death (green fluorescence) occurred primarily within the neuroepithelium. (G) qPCR (H) and Western blot analyses demonstrated the levels of *tp53* and Tp53 were increased respectively in 36 hpf and 5 dpf *polr1c*^-/-^ and *polr1d*^-/-^ mutant embryos compared to controls. α-tubulin was used as a loading control. * = p < 0.05 and error bars represent 95% confidence intervals.

In order to validate our hypothesis and determine the mechanistic basis underlying the apparent reduction of NCC in *polr1c*^-/-^ and *polr1d*^-/-^ mutant embryos, we tested whether *polr1c* and *polr1d* played functional roles in cell survival and/or proliferation. Using TUNEL staining as a marker of apoptosis, we observed increased cell death in 24 hpf *polr1c*^-/-^ and *polr1d*^-/-^ mutant embryos, particularly in the cranial region and along the dorsal aspect of the embryo (Fig 6A–6C). Transverse sections of TUNEL stained embryos revealed that the majority of cell death was localized within the neural tube or neuroepithelium, the dorsal-most regions of which contains NCC progenitors and pre-migratory NCC (Fig 6D–6F). Thus the reduced NCC population and subsequent pharyngeal arch hypoplasia observed in *polr1c*^-/-^ and *polr1d*^-/-^ mutant embryos, occurs at least in part due to pre-migratory NCC progenitor apoptosis.

However, it was important to determine whether cell death was also occurring in migrating NCC, which could also contribute to pharyngeal arch hypoplasia. Therefore, we bred *sox10*:*gfp* which labels migratory NCC, into the background of *polr1c*^-/-^ and *polr1d*^-/-^ mutant zebrafish and stained for apoptosis with TUNEL. We observed no significant co-localization of TUNEL with *sox10*:*gfp* at 24 or 48 hpf (S4 Fig). These results demonstrate that *polr1c* and *polr1d* loss-of-function specifically affects the viability of neuroepithelial cells in 24 hpf embryos. Thus, elevated apoptosis diminishes the pool of pre-migratory NCC, which leads to a reduced population of migrating NCC, resulting in pharyngeal arch hypoplasia and consequently cranioskeletal anomalies.

p53 is a well-known mediator of cell death underlying the pathogenesis of neurocristopathies and ribosomopathies [12]. We therefore hypothesized that the neuroepithelial apoptosis observed in *polr1c*^-/-^ and *polr1d*^-/-^ mutant embryos would also be p53-dependent. Quantitative RT-PCR (qPCR) revealed a significant increase in *tp53* transcript levels in 36 hpf mutant embryos (Fig 6G). *polr1c*^-/-^ embryos exhibited an approximately 6-fold higher level of *tp53* compared to control siblings while *polr1d*^-/-^ embryos displayed an approximately 4-fold higher level. In addition, Western blot analysis also revealed a substantial increase in the levels of Tp53 in 5 dpf mutant embryos compared to controls (Fig 6H). Collectively, these results suggested that the diminishment of migrating NCC in *polr1c*^-/-^ and *polr1d*^-/-^ mutant embryos, which occurs as a consequence of neuroepithelial cell death, was Tp53-dependent.

Cell and tissue hypoplasia can occur in response to decreased proliferation as well as increased apoptosis. Hence, as a further step towards understanding the roles of *polr1c* and *polr1d* during embryogenesis, it was important to determine whether cell proliferation was also affected in *polr1c*^-/-^ and *polr1d*^-/-^ mutant embryos. Therefore, we examined control, *polr1c*^-/-^ and *polr1d*^-/-^ mutant zebrafish in which migrating NCC were labeled with *sox10*:*gfp* and performed co-staining with the mitotic marker phospho-histone H3 (pHH3) to label proliferating cells. While overall pHH3 staining appeared to be similar between control and mutant embryos at 24 hpf (S5A–S5C Fig) and 36 hpf (S5D–S5F Fig; quantification in J), the proportion of proliferating cells within the NCC-derived pharyngeal arch 1 and 2 mesenchyme was considerably reduced in *polr1c*^-/-^ and *polr1d*^-/-^ embryos (S5G–S5I Fig and S5K Fig). Indeed only 6.7% of *sox10*:*gfp* labeled NCC in *polr1c*^-/-^ embryos co-labelled with pHH3 compared to 14% in control siblings. Thus, quantification of pHH3 positive NCC within the pharyngeal arches revealed that proliferation in *polr1c*^-/-^ embryos was reduced by as much as 50% compared to controls (S5K Fig). Furthermore, the rates of proliferation were similar between *polr1c*^-/-^ and *polr1d*^-/-^ embryos. Thus *polr1c* and *polr1d* loss-of-function diminishes the proliferation capacity of migrating NCC that colonize the pharyngeal arches. Taken together, our analyses demonstrate that Tp53-dependent apoptotic elimination of pre-migratory NCC, combined with decreased NCC proliferation, collectively results in fewer migrating NCC in *polr1c*^-/-^ and *polr1d*^-/-^ embryos compared to control siblings. This reduction in the number of migrating NCC and ensuing smaller pharyngeal arches can account for the hypoplasia of craniofacial cartilages observed in 5 dpf *polr1c*^-/-^ and *polr1d*^-/-^ mutant zebrafish.

### Mutations in *polr1c* and *polr1d* disrupt ribosome biogenesis

rRNA transcription accounts for up to 60% of all cellular transcription in eukaryotes and is a considered a rate-limiting step of ribosome biogenesis [2]. Furthermore, deficient ribosome biogenesis and nucleolar stress is associated with p53-dependent apoptosis [24]. Therefore we hypothesized that *polr1c* and *polr1d* loss-of-function should lead to diminished rRNA transcription and perturbed ribosome biogenesis underpinning the activation of Tp53-dependent apoptosis in *polr1c*^-/-^ and *polr1d*^-/-^ mutant embryos.

Ribosome biogenesis begins with transcription of the 47S precursor rRNA by RNA Pol I and 5S rRNA by RNA Pol III. The 47S rRNA contains a 5’ externally transcribed sequence (ETS) and two internally transcribed sequences (ITS1 and ITS2), which separate the 18S, 5.8S, and 28S rRNA sequences. The 5’ETS, ITS1, and ITS2 are subsequently cleaved from the 47S transcript as part of the processing that generates the mature 18S, 5.8S, and 28S rRNAs during ribosome biogenesis. The 5’ETS, ITS1, and ITS2 transcripts can be used as an estimate of 47S transcription [25], and may provide a more sensitive indicator of perturbations in rRNA synthesis than the steady-state levels of mature 18S or 28S rRNAs [26].

**Fig 7:**
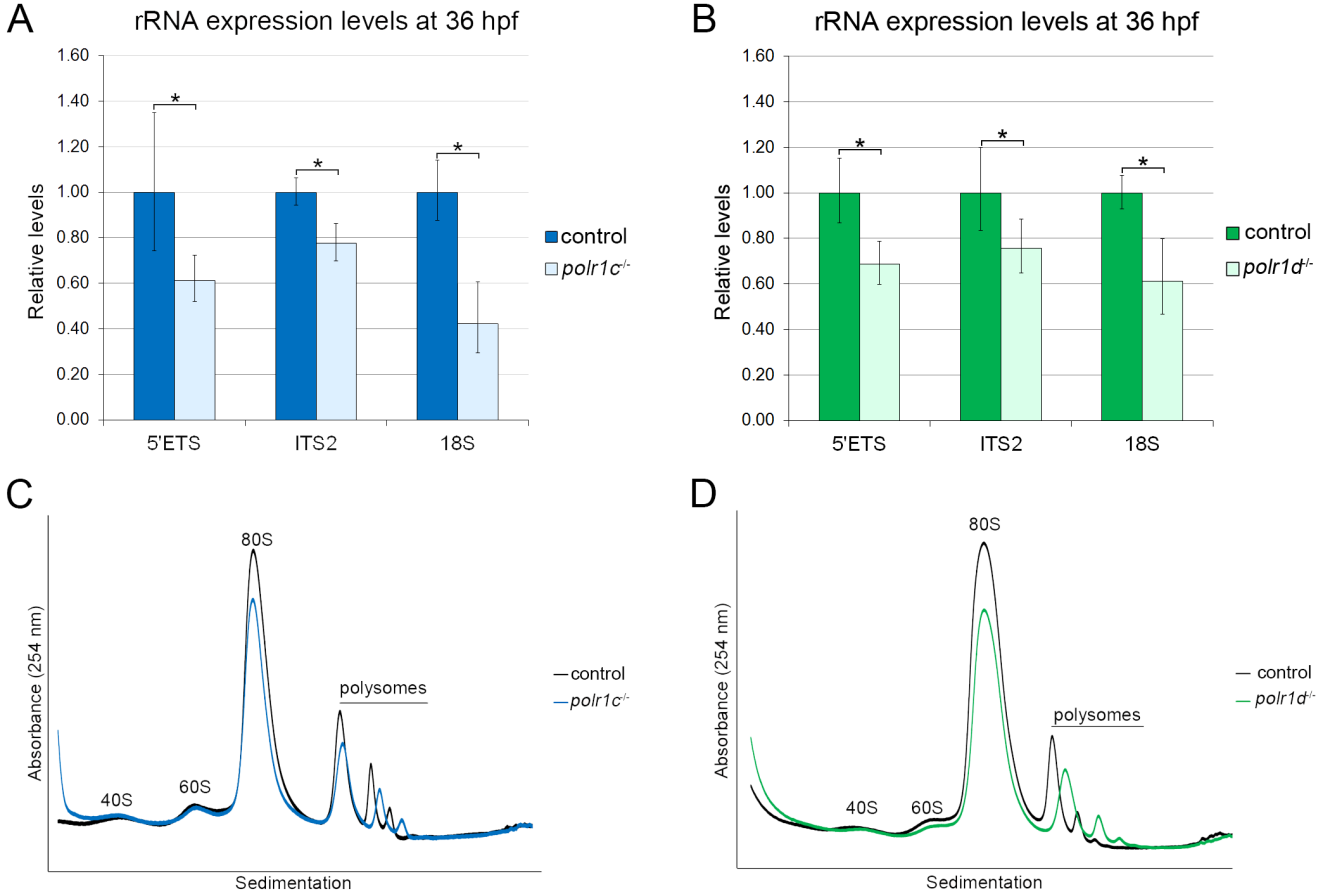
Ribosome biogenesis is disrupted in *polr1c* and *polr1d* mutant embryos. (A, B) qPCR quantification of 47S rRNA production. (A) *polr1c*^-/-^ mutants exhibit reduced levels of 5’ETS (39%), ITS2 (23%) and 18S rRNA (58%) compared to controls. (B) *polr1d*^-/-^ mutants similarly exhibit reduced levels of 5’ETS (25%), ITS2 (39%), and 18S rRNA (32%) compared to controls. (C, D) Polysome profiling shows decreased 80S and polysome peaks in *polr1c*^-/-^ (C) and *polr1d*^-/-^ (D) mutant embryos.

To validate our hypothesis, we evaluated rRNA transcription by quantifying the levels of 5’ETS, ITS2, and 18S rRNAs by qPCR. We observed a significant reduction of the 5’ETS, ITS2, 18S rRNA transcripts in *polr1c*^-/-^ and *polr1d*^-/-^ mutant embryos compared to control siblings (Fig 7A and 7B). At 36 hpf, the 5’ETS is reduced by 38% in *polr1c*^-/-^ mutants and 32% in *polr1d*^-/-^ mutants whereas ITS2 is reduced by 23% in *polr1c*^-/-^ mutants and 25% in *polr1d*^-/-^ mutants, relative to control embryos. The levels of 18S rRNA, which reflect the activity of both the precursor 47S transcript as well as the processed fully mature 18S rRNA, were also considerably diminished in *polr1c*^-/-^ and *polr1d*^-/-^ mutant embryos. In fact, *polr1c*^-/-^ mutant embryos exhibited a 58% reduction in the levels of 18S compared to controls. *polr1d*^-/-^ mutant embryos exhibited a similar reduction of about 39% compared to controls. To understand the impact of *polr1c* and *polr1d* mutations on RNA Pol III function in ribosome biogenesis in addition to their function as a part of RNA Pol I, we investigated the levels of 5S rRNA. No significant changes were observed in the levels of 5S rRNA in *polr1c*^-/-^ and *polr1d*^-/-^ mutant embryos (S1 Fig). Taken together, our data demonstrates as predicted, that 47S rRNA transcription is reduced in *polr1c*^-/-^ and *polr1d*^-/-^ mutant embryos and furthermore, that the disruption of rRNA synthesis primarily occurs as a result of perturbed RNA Pol I function.

Given that 47S rRNA transcription is considered a rate-limiting step during ribosome biogenesis [2], we hypothesized that reduced rRNA transcription in *polr1c*^-/-^ and *polr1d*^-/-^ mutant embryos would result in an overall reduction in ribosome biogenesis. We evaluated ribosome biogenesis in 3 dpf *polr1c*^-/-^ and *polr1d*^-/-^ mutant embryos and control siblings through polysome profiling (Fig 7C and 7D). The polysome profiles revealed similar sized 40S (small subunit) and 60S (large subunit) peaks in mutant embryos compared to controls. This indicates that the ratio of small and large subunit production was not affected in *polr1c*^-/-^ and *polr1d*^-/-^ mutant embryos. However, there were reductions in the 80S peak in both *polr1c*^-/-^ and *polr1d*^-/-^ mutant embryos compared to controls, which is indicative of a deficiency in the production or assembly of functional 80S ribosomes. The polysome peaks were shorter and slightly broader in mutant embryos compared to controls, highlighting an overall decrease in ribosome biogenesis in *polr1c*^-/-^ and *polr1d*^-/-^ mutant embryos, which is consistent with diminished production of 47S rRNA (Fig 7A and 7B). Collectively these data suggest that decreased rRNA transcription and perturbed ribosome biogenesis contribute to the Tp53-dependent neuroepithelial apoptosis and diminished pool of migrating NCC that occurs in association with craniofacial cartilage hypoplasia in *polr1c*^-/-^ and *polr1d*^-/-^ mutant zebrafish.

### Genetic inhibition of *tp53* ameliorates cranioskeletal anomalies in *polr1c* and *polr1d* mutants

Our data demonstrated a correlation between deficient ribosome biogenesis, Tp53-dependent cell death, and craniofacial anomalies characteristic of TCS in *polr1c*^-/-^ and *polr1d*^-/-^ mutant zebrafish. Consequently, we hypothesized that inhibition of Tp53 would suppress neuroepithelial apoptosis and prevent the pathogenesis of craniofacial anomalies. To test our hypothesis, we crossed the *tp53*^M214K/M214K^ allele into the background of *polr1c*^-/-^ and *polr1d*^-/-^ mutant zebrafish in an effort to inhibit Tp53 function. The *tp53*^M214K/M214K^ allele, hereafter referred to as *tp53*^-/-^, carries a mutation within the DNA-binding domain of *tp53* [27] that disrupts its ability to initiate the transcription of downstream target genes.

**Fig 8:**
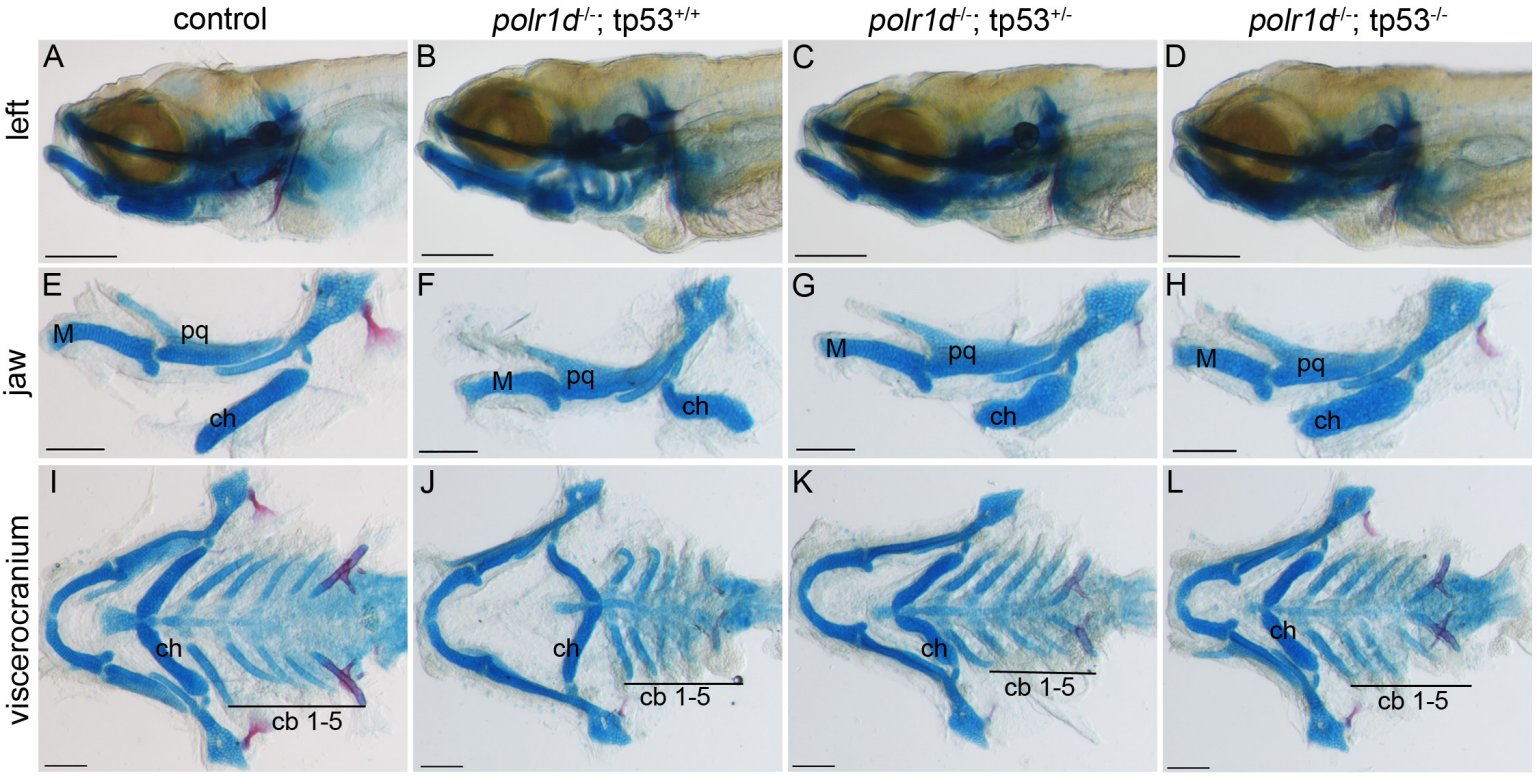
*tp53* inhibition ameliorates cartilage anomalies in *polr1d*^-/-^mutant embryos in a dosage-dependent manner. (A-D) Alcian blue staining of cartilage in an allelic series of *polr1d* and *tp53* mutant embryos. Dosage-dependent improvement in cartilage development is particularly noticeable in the jaw (E-H), elements of the viscerocranium (I-L), and more specifically the ceratohyal (G-L). Abbreviations: M, Meckel’s cartilage; pq, palatoquadrate; ch, ceratohyal; cb, ceratobranchial. Scale bar = 200 μm.

Consistent with our prediction, TUNEL staining revealed reduced levels of cell death in 24 hpf *polr1c*^-/-^; *tp53*^-/-^ and *polr1d*^-/-^; *tp53*^-/-^ embryos compared to *polr1c*^-/-^ and *polr1d*^-/-^ siblings (S6 Fig; S7 Fig). Transverse sections further demonstrated that the specific reduction of cell death within the neuroepithelium of *polr1c*^-/-^ and *polr1d*^-/-^ embryos was dose-dependent for *tp53* (S6E–S6H Fig; S7E–S7H Fig). Consistent with these results, we also observed a similar *tp53* dose-dependent rescue of cranial cartilage formation in *polr1c*^-/-^ (S8 Fig) and *polr1d*^-/-^ mutant embryos (Fig 8). Removal of one copy of *tp53* improved jaw development (Fig 8F and 8G) and patterning of the viscerocranium including the ceratohyal and ceratobranchial cartilages (Fig 8J and 8K) in *polr1c*^-/-^ and *polr1d*^-/-^ embryos. For example, although the ceratohyal remained smaller compared to control siblings, its polarity was restored to normal. Similarly, the ceratobranchials were larger and displayed more organized stacking. Removal of both copies of *tp53*, and thereby complete inhibition of Tp53, rescued the craniofacial phenotype to an even greater degree (Fig 8H and 8L). For example, the ceratohyal was more elongated in *polr1d*^-/-^; *tp53*^-/-^ embryos compared to *polr1d*^-/-^; *tp53*^+/-^ embryos.

To provide a more detailed analysis of the ability of Tp53 inhibition to prevent the pathogenesis of craniofacial anomalies in *polr1c*^-/-^ and *polr1d*^-/-^ mutant embryos, we classified the cranial cartilage phenotypes as severe, mild, or wild-type, and quantified the proportion of embryos commensurate with each category (S9 Fig). The severe category included mutants with a hypoplastic ceratohyal of reversed polarity, whereas mild mutants exhibited a forward projecting but still hypoplastic ceratohyal. The wild-type category denoted embryos with a ceratohyal of relatively normal size and polarity, commensurate with wild-type embryos. Loss of one copy of *tp53* prevented craniofacial anomalies in 19% of *polr1c*^-/-^; *tp53*^+/-^ and 24% of *polr1d*^-/-^; *tp53*^+/-^ embryos as each of these embryos developed with a wild-type phenotype. At the same time we observed a concomitant reduction in the number of *polr1c*^-/-^; *tp53*^+/-^ and *polr1d*^-/-^; *tp53*^+/-^ embryos that were classified as severe to 32% and 22% respectively, with the remainder presenting with a mild phenotype. Consistent with *tp53* dose-dependency, removal of both copies substantially improved the efficacy of rescue. The percentage of *polr1c*^-/-^; *tp53*^-/-^ and *polr1d*^-/-^; *tp53*^-/-^ embryos that exhibited a wild-type phenotype dramatically increased to 62% and 35% respectively. Furthermore, the percentage of *polr1c*^-/-^; *tp53*^-/-^ and *polr1d*^-/-^; *tp53*^-/-^ embryos that exhibited the severe phenotype was concomitantly reduced to 9.5% and 0% respectively, with the remainder displaying a mild phenotype.

Despite the considerable improvement in formation and patterning of the cranial cartilages in *polr1c*^-/-^; *tp53*^-/-^ and *polr1d*^-/-^; *tp53*^-/-^ embryos, the skeletal elements generally remained slightly smaller overall relative to control siblings. Furthermore, although Tp53 inhibition was sufficient to suppress neuroepithelial cell death and dramatically prevent cranioskeletal anomalies in *polr1c*^-/-^ and *polr1d*^-/-^ mutant embryos, this was still insufficient to rescue their long-term viability. *polr1c*^-/-^; *tp53*^-/-^ and *polr1d*^-/-^; *tp53*^-/-^ mutant zebrafish die around 10 dpf, which is very similar to *polr1c*^-/-^ and *polr1d*^-/-^ mutant zebrafish. Nonetheless, our results demonstrate that *polr1c* and *polr1d* play critical roles in rRNA transcription and ribosome biogenesis during embryogenesis and particularly in craniofacial development. Furthermore, we have established *polr1c*^-/-^ and *polr1d*^-/-^ mutant zebrafish as new models of TCS. *polr1c* and *polr1d* loss-of-function perturbs ribosome biogenesis which leads to Tp53-dependent neuroepithelial apoptosis, a diminished population of migrating NCC with reduced proliferation capacity, pharyngeal arch hypoplasia, and consequently craniofacial anomalies. Tp53 inhibition can suppress neuroepithelial apoptosis and substantially rescue cranioskeletal development in *polr1c*^-/-^ and *polr1d*^-/-^ mutant embryos providing a potential avenue for the therapeutic prevention of TCS.

## Discussion

Congenital craniofacial anomalies account for approximately one-third of all birth defects in newborn babies [28] and to date more than 700 distinct syndromes have been reported [29]. Craniofacial disorders are typically described and classified according to the extent of alterations to the craniofacial skeleton, which is derived primarily from NCC [17]. Most craniofacial anomalies are therefore attributed to defects in NCC development. In order to develop therapeutic avenues for minimizing or preventing craniofacial anomalies, it is essential to understand the precise etiology and pathogenesis of individual malformation syndromes. This requires a thorough understanding of (i) the normal signals and mechanisms that regulate NCC formation, survival, migration and differentiation; and (ii) the functional developmental roles played by genes that are mutated in association with the etiology of specific disorders.

Facial dysostosis describes a set of clinically and etiologically heterogeneous congenital craniofacial anomalies that encompass maxillary, malar and mandibular hypoplasia, together with cleft palate, and/or ear defects [30]. Facial dysostosis can be subdivided into acrofacial dysostosis and mandibulofacial dysostosis. Acrofacial dysostosis presents with similar craniofacial anomalies to those observed in mandibulofacial dysostosis but with the addition of limb defects. Several distinct mandibulofacial dysostosis syndromes have been documented, with the most well-known and best understood being TCS [31]. TCS, which is also known as mandibulofacial dysostosis and Franschetti-Zwahlen-Klein syndrome [32], is characterized primarily by hypoplasia of the facial bones, particularly the maxilla, mandible and zygomatic complex. In addition, the palate is often high-arched or frequently cleft [33,34].

*TCOF1* encodes a nucleolar phosphoprotein called Treacle, which promotes rDNA transcription via direct binding of upstream binding factor (UBF) and RNA Pol I in the nucleolus. *Tcof1* is broadly expressed throughout the mouse embryo during embryogenesis with elevated levels of activity in the neuroepithelium where it plays a vital role in cell survival. Analyses of a *Tcof1*^*+/-*^ mouse model of TCS determined that this disorder arises through extensive p53-dependent neuroepithelial apoptosis, together with a deficiency in the generation and proliferation of NCC, which are the precursors of the craniofacial skeleton [35–37]. Furthermore, *Tcof1* haploinsufficiency leads to deficient ribosome biogenesis [38] which provides the trigger for induction of p53-mediated apoptosis [39]. Consistent with this mechanism, genetic and pharmacological inhibition of p53 can suppress neuroepithelial apoptosis in *Tcof1*^*+/-*^ embryos and prevent the pathogenesis of craniofacial anomalies characteristic of TCS [37].

TCS is therefore considered to be both a neurocristopathy and ribosomopathy disorder. However, mutations in *TCOF1* do not account for all individuals diagnosed with TCS. Whole exome sequencing of individuals with TCS that lacked a mutation in *TCOF1*, subsequently revealed causative mutations in *POLR1C* and *POLR1D* [40]. In contrast to *TCOF1*, the mutations identified to date in *POLR1C* are all autosomal recessive [40]. However, similar to *TCOF1*, mutations in *POLR1C* perturb its function as a part of RNA Pol I [41]. Analyses in HeLa cells revealed that RNA Pol I targeting to the nucleolus was reduced in association with *POLR1C* mutations in the pathogenesis of TCS. In contrast, no effect on the assembly or function of Pol III was observed. More recently, recessive mutations in *POLR1C* have been found to cause leukodystrophy, or degeneration of white matter in the brain, and interestingly, these mutations alter POLR1C function specifically as a part of RNA Pol III. Leukodystrophy associated mutations in *POLR1C* perturb Pol III assembly and occupancy at Pol III promoters but not Pol I assembly or occupancy at the rDNA promoter. With respect to *POLR1D*, at least 17 distinct mutations have been described, and similar to *TCOF1*, they mainly elicit their effect in an autosomal dominant manner [7,40,42,43]. However, similar to *POLR1C*, recessive mutations in *POLR1D* have also been identified in association with TCS, but to date none of these have been linked to leukodystrophy.

In contrast to our understanding of the role of *TCOF1* during embryogenesis and in the etiology and pathogenesis of TCS, there is a paucity of information about *POLR1C* and *POLR1D*. Therefore, we set out to explore the functional roles of *polr1c* and *polr1d* during embryogenesis and more specifically in craniofacial development in an effort to better understand the pathogenesis of TCS and the tissue-specificity of this ribosomopathy. We discovered that *polr1c* and *polr1d* are dynamically and spatiotemporally expressed during zebrafish embryogenesis. In particular, *polr1c* and *polr1d* exhibit elevated levels of expression in specific craniofacial tissues from as early as 24 hpf. Consistent with this pattern of activity, zebrafish with mutations in *polr1c* and *polr1d* present with cranioskeletal hypoplasia that mimics TCS in humans. Our studies also showed that reduced rRNA production in *polr1c*^-/-^ and *polr1d*^-/-^ mutant embryos led to induction of Tp53 dependent neuroepithelial apoptosis. This in turn resulted in fewer migrating NCC, which exhibited decreased proliferation capacity during colonization of the pharyngeal arches. Consequently the pharyngeal arches in 36 hpf *polr1c*^-/-^ and *polr1d*^-/-^ mutant zebrafish were hypoplastic, and this manifested as small and often malformed craniofacial cartilages in 5 dpf fish. Consistent with all of this data, genetic inhibition of *tp53* ameliorated the cranioskeletal malformations in *polr1c*^-/-^ and *polr1d*^-/-^ mutant embryos in a dose-dependent manner.

Collectively, our data demonstrates that *polr1c* and *polr1d* are spatiotemporally expressed and play critical roles in rRNA transcription and ribosome biogenesis during zebrafish embryogenesis. Furthermore, *polr1c* and *polr1d* are essential for neuroepithelial cell survival and NCC proliferation during zebrafish craniofacial development. Moreover, these data are consistent with analyses of craniofacial development in *Tcof1*^*+/-*^ mouse embryo models of TCS. Thus, TCS is caused by mutations in three distinct genes involved in rRNA transcription: *TCOF1*, *POLR1C*, and *POLR1D* [10–13,30]. Here we have identified a common unifying cellular and biochemical mechanism underpinning the pathogenesis of TCS irrespective of whether its cause is associated with mutations in *TCOF1*, *POLR1C*, or *POLR1D*. Mutations in *TCOF1* account for about 80% of patients with TCS, while mutations in *POLR1C* and *POLR1D* account for only about 2% of the patients sequenced to date. This suggests that mutations in additional genes may also be causative for TCS.

Other subunits of RNA Pol I, or factors that interact with TCOF1, POLR1C, and POLR1D, make ideal candidates for an association with the etiology of TCS. Consistent with this idea, we recently identified mutations in the RNA Pol I subunit, *POLR1A*, in association with another ribosomopathy disorder, Acrofacial dysostosis, Cincinnati type [44]. POLR1A is the largest subunit of RNA Pol I and contains the active site of the polymerase [45,46]. Acrofacial dysostosis, Cincinnati type is characterized by hypoplasia of the zygomatic arches, maxilla, and mandible, with or without limb skeletal defects. Acrofacial dysostosis comprises a subgroup of facial dysostosis, and the craniofacial phenotype overlaps considerably with mandibulofacial dysostosis of which TCS is a prime example.

Similar to *polr1c* and *polr1d*, *polr1a* is also dynamically expressed during zebrafish embryogenesis, particularly with respect to craniofacial development [44]. Furthermore, *polr1a* loss-of-function also leads to perturbed rRNA transcription, decreased ribosome biogenesis and Tp53-dependent cell death, resulting in a deficiency of NCC derived skeletal precursor cells and consequently craniofacial anomalies. Thus the tissue-specific phenotypes that result from alterations in rRNA transcription and ribosome biogenesis, exhibit a common underlying mechanism. This is true not just for TCS and Acrofacial dysostosis, Cincinnati type, as deficient ribosome biogenesis induced p53-dependent cell death, and rescue by p53 inhibition, has also been observed for Diamond Blackfan anemia (DBA) and 5q- syndrome [12,47–51].

The spatiotemporally dynamic expression of *polr1a*, *polr1c*, and *polr1d* during zebrafish embryogenesis, particularly in craniofacial tissues, is consistent with their loss-of function phenotypes as well as with the etiology and pathogenesis of Acrofacial dysostosis, Cincinnati type and TCS, respectively. However, the tissue-specific activity and function of these RNA Pol I subunits is surprising given that rRNA transcription is considered one of the rate-limiting steps of ribosome biogenesis, and furthermore that ribosome biogenesis is a tightly regulated global process, thought to be integral to all cell growth and proliferation.

The similarity in dynamic expression and function for *polr1a*, *polr1c*, and *polr1d* lends support to the idea that different tissues have different threshold requirements for RNA Pol I activity and thus ribosome biogenesis during development. Consistent with this idea, cells with higher rates of ribosome biogenesis prior to RNA Pol I perturbation have been found more likely to undergo p53-induced apoptosis [52]. In contrast, cells with lower rates of ribosome biogenesis undergo cell cycle arrest. This raises the interesting possibility in the context of *Tcof1*, *polr1a*, *polr1c*, and *polr1d* mutant embryos, that neuroepithelial cells have a higher rate of rRNA transcription and ribosome biogenesis and are thus more likely to undergo apoptosis than other cell types with lower rates of rRNA transcription and ribosome biogenesis.

Our data suggests that there may well be differential or tissue specific levels of rRNA transcription during embryogenesis and/or that individual tissues may require distinct threshold levels of ribosome biogenesis for normal development and function. In the future, it will be important to quantify the relative levels of rRNA transcription and ribosome biogenesis in specific tissues during embryogenesis to determine whether there is an association between threshold levels and the effect of perturbation. Further evidence of tissue specific requirements for ribosome biogenesis comes from other ribosomopathies. Diamond-Blackfan anemia (DBA) for example is a ribosomopathy characterized primarily by disruptions of the erythroid precursor population but can also occur together with craniofacial and digit anomalies in some individuals. The majority of DBA cases result from mutations in ribosomal protein genes that encode protein constituents of either the 40S or 60S ribosomes [53] [54] [48,55,56].

The subtle differences in *polr1a*, *polr1c*, and *polr1d* expression and ensuing loss-of-function phenotypes raises the intriguing possibility that perhaps the subunit composition of RNA Pol I may be spatiotemporally dynamic, or alternatively, that translation may occur in a tissue specific manner in the form of specialized ribosomes. In agreement with this idea, *Rpl38*^*-/-*^ mouse embryos exhibit tissue defects in cranial and axial skeleton development [57]. These defects were found to be specifically associated with altered *Hox* gene expression. Thus, mutation of *Rpl38* did not affect global protein synthesis but rather specifically impacted the translation of *Hox* genes, implying a tissue specific role for Rpl38 in ribosome biogenesis and translation. Bent Bone Dysplasia syndrome (BBDS) provides further evidence for both tissue threshold sensitivity as well as differential control of ribosome biogenesis by lineage-specific factors [58]. BBDS is characterized by bent long bones, underdeveloped clavicles and pubic bone together with poor mineralization of the skull, and is associated with mutations in *FGFR2*. The *FGFR2* mutations in BBDS activate rDNA transcription and alter osteoblast differentiation. In preosteoblasts, nucleolar FGFR2 represses RUNX2, which functions as a transcription factor to promote osteoblast differentiation by repressing rDNA transcription at rDNA promoters [59]. These studies suggest not only a specific link between ribosome biogenesis and bone formation but also that ribosome biogenesis may be a mechanism for coordinating proliferation and cell fate. It will be important to explore whether *polr1c* and *polr1d* also interact with factors such as Runx2 and function specifically in osteoblast differentiation.

Numerous rRNA cleavage and processing events together with ribosome co-factors collectively offer considerable opportunities for the differential or spatiotemporally specific regulation of ribosome biogenesis during embryogenesis. The similarity of expression patterns for *polr1a*, *polr1c*, and *polr1d* lends strong support to the idea that particular tissues such as neuroepithelial cells and neural crest cell progenitors require high levels of RNA Pol I activity during embryogenesis. However, the roles of polr1c and polr1d as a part of RNA Pol III remain to be fully investigated. Our results showed that 5S rRNA transcription is not affected in the *polr1c*^-/-^ mutant zebrafish. Furthermore, other studies have revealed that mutations in *POLR1C* that are associated with the etiology and pathogenesis of TCS do not impact RNA Pol III function [41]. Nonetheless, RNA Pol III performs several important functions in cells including transcription of the 5S rRNA as well as tRNAs and ncRNAs [60], and yet the roles of RNA Pol III during embryogenesis remain poorly understood. In the future, it will therefore be important to determine whether specific mutations exert distinct effects on RNA Pol I versus RNA Pol III function in different tissues and in association with the different phenotypes characteristic of TCS and leukodystrophy. It is also important to be cognizant of other possibilities such as functions for RNA Pol I and III subunits in processes other than rRNA transcription and ribosome biogenesis as has recently been shown for *TCOF1*/Treacle [61,62].

In summary, we described the spatiotemporal activity and functional roles of polr1c and polr1d during embryogenesis and particularly in craniofacial development. We discovered that polr1c and polr1d play important functions in rRNA transcription and furthermore that *polr1c* and *polr1d* loss-of-function results in tissue-specific phenotypes, including craniofacial cartilage anomalies that mimic TCS in humans. Moreover, inhibition of Tp53 function was able to ameliorate cranioskeletal anomalies in *polr1c*^-/-^ and *polr1d*^-/-^ mutant zebrafish. Collectively our data provides a unifying cellular and biochemical mechanism underlying the pathogenesis of TCS irrespective of whether *TCOF1*, *POLR1C*, or *POLR1D* is mutated. The tissue-specific phenotypes we observed in *polr1c*^-/-^ and *polr1d*^-/-^ mutant zebrafish augment a growing body of work suggesting that rRNA transcription and ribosome biogenesis are dynamically regulated during embryogenesis. Our results therefore provide new insights into the tissue specific roles of RNA Pol I during development and in the etiology and pathogenesis of TCS.

## Materials and Methods

### Ethics statement

Adult zebrafish (*Danio rerio*) were housed and maintained in the Stowers Institute Zebrafish Facility according to IACUC standards and as detailed in Protocol # 2015–0138 which was approved on 6/24/2015.

### Zebrafish

Zebrafish embryos were raised at 28.5°C and staged according to Kimmel *et al*., 1995 [63]. When necessary, 1-Phenyl-2-thiourea (0.002%) was added to the embryo media to prevent pigment development. *polr1c*^hi1124Tg^, and *polr1d*^hi2393Tg^ zebrafish were maintained as heterozygotes and incrossed to generate homozygous mutant embryos. The *polr1c* and *polr1d* heterozygous mutant lines were crossed with additional available reporter lines including Tg(*fli1a*:*egfp*), referred to as *fli1a*:*egfp*, and Tg(7.2kb-*sox10*:*gfp*), referred to as *sox10*:*gfp*, as well as the *tp53*^M214K^ line. PCR was conducted on *polr1c* and *polr1d* adults and embryos for the presence of the insertional mutation. The *polr1c* wild type allele was detected using the primers forward 5’-CTATTGCTTTTGTCGCATAAAGCG-3’ and reverse 5’-CTCCAGTGTGTTTTCATCTGAAC-3’. The *polr1c* mutant allele was detected using the primers forward 5’-CTATTGCTTTTGTCGCAT AAAGCG-3’ and reverse 5’-GCTAGCTTGCCAAACCTACAGGT-3’. The *polr1d* wild type allele was detected using primers forward 5’-CAGTCACAACGTGCGACATGC-3’ and reverse 5’-GGTAAACGAGTTGATTTACGCATTG-3’ and the mutant allele detected using forward 5’-CAGTCACAACGTGCGACATGC-3’ and reverse 5’-GCTAGCTTGCCAAACCTACAGGT-3’.

### Phenotype analysis

#### Live imaging

Embryos were anaesthetized with MS-222 and mounted in 2% methyl cellulose while submerged in E2 media. Embryos were imaged using a Leica MZ16 microscope equipped with a Nikon DS-Ri1 camera and NIS Elements BR 3.2 imaging software. When appropriate, manual Z stacks were taken and the images were assembled using Helicon Focus software.

#### Skeletal stain

Alcian blue and Alizarin red staining was completed according to Walker and Kimmel [64]. Tails were removed after staining for genotyping. Embryos were cleared into glycerol and dissected for flat mounted images. Imaging was completed on the same system listed above.

#### In situ hybridization

Regions of *polr1c and polr1d* were amplified from cDNA using the primers forward 5’-caacgtggatgaaattcgtg-3’ and reverse 5’-caccttccttccgttca cat-3’ for *polr1c* and primers forward 5’-ggctgagcttggacagaaac-3’ and reverse 5’-cagtgtccacatgc tcacaa-3’ for *polr1d*. These amplified regions were cloned into the TOPO II vector (Invitrogen). The vector was used to generate both sense and anti-sense probes for in situ hybridization. In situ hybridization was completed according to standard protocols. Briefly, embryos were permeabilized with Proteinase K, and hybridized in probes diluted to 2.5 ng/μl overnight at 66–68°C. The probe was removed and embryos were washed and blocked for a minimum of 1 hour prior to incubation in AP-Fab (1:5000, Roche). Signal was detected using NBT/BCIP and the development reaction was stopped upon the presence of any background signal in the sense probe controls. Embryos were cleared through a glycerol series and imaged on the system listed previously.

#### Immunostaining and TUNEL

Whole-mount immunostaining was completed according to Westerfield, 2000 (The Zebrafish Book) using primary antibodies against GFP (1:500, Invitrogen), Zn-8 (1:250, DSHB), and pHH3 (1:2000, Millipore). Fluorescent secondary antibodies, either Alexa-488 or Alexa-546 (1:500, Invitrogen) were used for detection. TUNEL was completed according to Crump *et al*. 2004 [65] with slight modifications. Embryos were permeabilized overnight in methanol at -20°C and incubated for 1 hour at 37°C in either the Fluorescein or the TdT/TMR red reaction. Embryos were imaged using a Zeiss upright 700 confocal microscope and images were captured and processed using Zen software.

### Molecular analysis

#### Western blot

Protein samples of 100 fish/sample were collected at 5 dpf. Embryos were homogenized and suspended in sample buffer (100 mM Tris pH 6.8, 4% SDS, 20% glycerol, 10% β-mercaptoethanol) and used for Western blotting according to standard protocols (The Zebrafish Book, zfin.org). Primary antibodies used were α-tubulin (1:10000, Sigma) and Zfish Tp53 (1:500, Anaspec). Blots were incubated with ECL-Horseradish peroxidase secondary antibodies (1:5000; GE Healthcare), followed by reaction with Amersham ECL Prime Western Blotting Detection Reagent (GE Healthcare), according to the manufacturer’s instructions. Western blots were developed on autoradiography film (MidSci), and films were scanned and quantified using ImageJ.

#### qPCR

RNA was collected from zebrafish embryos using the Qiagen miRNeasy Micro Kit, treated with DNase, and tested for quality and concentration on an Agilent 2100 Bioanalyzer. Equal amounts of RNA were used to synthesize cDNA for qPCR with the Superscript III kit (Invitrogen) using random hexamer primers. Primers used for qPCR were *polr1c* forward 5’-GGCTGAGGTTCCAACAA-3’ and reverse 5’-GACGAGGGTCTGCTTTAAT -3’; *polr1d* forward 5’-GATACAGACGCGAGATGG -3’ and reverse 5’-GCTCCTTGAATTCTTTCATCC-3’; *tp53* forward 5’-CGAGCCACTGCCATCTATAAG-3’ and reverse 5’-TGCCCTCCACTC TTATCAAATG-3’. rRNA primer sequences were obtained from Azuma *et al*., 2006 [25], 5S rRNA primers were forward 5’-CCATACCACCCTGGAAAT-3’ and reverse 5’-CTCCCATCCAAGTACTAACC-3’. Controls used were *β-actin*, *canx*, and *ef1*. Primers for these were *β-actin* forward 5’-ACATCAGGGAGTGATGGTTGGCAT-3’ and reverse 5’-AGTCACAA TACCGTGCTCAATGGG-3’; *canx* forward 5’-ACGATACCGCAGAGAATGGAGACA-3’ and reverse 5’–TCCTGTTTCTGGGAGACCTCCTCA-3’; *ef1* forward 5’–CTCAAATGGCATGGA TGTTGCCCA-3’ and reverse 5’- GGTCTTGGTTTGCGCACTTTGGTT-3’. Assays were validated using a dilution series of cDNA from 36 hpf AB embryos with concentrations ranging from 0.1 ng/μl to 50 ng/μl. The results of the assay validation were used to determine the reaction efficiency and optimal cDNA concentration. PerfeCTa (Quanta Biosciences) reaction mix and the ABI 7900HT real time PCR cycler were used measure cDNA amplification. Five biological replicates were run in technical triplicate for each experiment. No template and no reverse-transcriptase samples were run as negative controls. Data was analyzed using Biogazelle software and efficiencies were adjusted to 1.57 for the 5’ETS and 1.50 for ITS2 based on our assay validation experiments. The Mann-Whitney test was used to determine statistical significance.

#### Polysome profiling

150 embryos per sample were collected at 3 dpf and identified by phenotype. Embryos were deyolked and rinsed with ice cold PBS and then dissociated in ice-cold lysis buffer (10mM Tris-HCl, 5 mM MgCl2, 100 mM KCL, 1% TritonX-100, 2 mM DTT, 100ug/ml cycloheximide, 200U/ml RNasin (Promega), and protease inhibitor (Sigma)). Homogenized zebrafish were then centrifuged at 15000 x g at 4°C for 10 minutes and the supernatant of the zebrafish lysate was kept for analysis. Lysates were loaded onto a 10–50% sucrose gradient prepared in 20 mM HEPES-KOH pH 7.4, 5 mM MgCl_2_, 100 mM KCl, 2mM DTT, 20 U/mL RNasin (Promega), and 100 ug/mL cycloheximide. The gradients were ultra-centrifuged at 4°C in an SW-41 Ti rotor (Beckman) at 40,000 rpm for 2 hours. To evaluate UV absorbance profiles, each gradient was passed through a UA-6 absorbance reader system (Teledyne ISCO) using a syringe pump (Brandel). The absorbance at 254 nm was recorded using WinDaq data acquisition software (DATAQ INSTRUMENTS) and the profiles were plotted in Microsoft Excel.

#### Image quantification

To quantify regions of interest in confocal images, IMARIS software was used. The surfaces generated from the images used the automated settings. Within the region of interest determined by the surface, the software was used to calculate measurements including area and volume. Cell counts were determined within the region of interest using the spots tool. For cell counts per volume, the surfaces were generated with smoothing set to 25 and a threshold of 15, while the quality in the spots tool was set to 9.0. A two-tailed Student’s t-test was used to determine statistical significance.

## Supporting Information

S1 Fig qPCR shows significantly reduced expression of *polr1c* and *polr1d* at 36 hpf. (A) *polr1c* reduced by approximately 80% in homozygous mutant embryos while levels of 5S rRNA remain unchanged. (B) *polr1d* is reduced by approximately 97% in homozygous mutant embryos while 5S rRNA levels are unchanged. * = p < 0.05 and error bars represent 95% confidence intervals.
(TIF)

S2 Fig *polr1c* and *polr1d* mutant embryos have craniofacial cartilage anomalies at 9 dpf. (A-C) Alcian blue and Alizarin red staining reveals diminished cartilage and bone formation in *polr1c* and *polr1d* mutant embryos. (D-F) Dissection of the viscerocranium revealed mispatterning of the ceratohyal (ch), and hypoplasia of the ceratobranchial cartilages (cb) in *polr1c* and *polr1d* mutants. There is also hypoplasia of bone elements including the opercles (op) and pharyngeal teeth. (G-H) Dissection of the neurocranium reveals reduced ossification of the parasphenoid (ps) in mutant embryos. Scale bar = 200 μm.
(TIF)

S3 Fig Neural plate formation in 11 hpf embryos. (A-C) *polr1c*^-/-^ and *polr1d*^-/-^ mutant embryos show similar expression of *sox2* compared to controls as revealed by in situ hybridization. This indicates that neural plate formation, a precursor to formation of neural crest cells, occurs in mutant embryos.
(TIF)

S4 Fig The migratory NCC population is not undergoing apoptosis in *polr1c* and *polr1d* mutant embryos. (A-D) TUNEL staining in *sox10*:*gfp* embryos shows increased cell death in mutant embryos which does not co-localize with the migratory NCC population. At 24 hpf, increased cell death can be seen in the neuroepithelial region of *polr1c*^-/-^ and *polr1d*^-/-^ embryos. (E-F) At 48 hpf, increased cell death can be observed in regions of the brain and eye of mutant embryos, but not within the pharyngeal arches. Scale bar = 200 μm.
(TIF)

S5 Fig Proliferation within pharyngeal arches 1 & 2 is reduced. (A-C) similar levels of pHH3 staining are present in control, *polr1c*^-/-^ and *polr1d*^-/-^ embryos at 24 hpf. (D-F) Proliferation at 36 hpf also occurs globally at broadly similar levels in controls and mutant embryos, but there differences in the number of pHH3+ cells within pharyngeal arches 1 and 2. (G-I) Magnified views of pharyngeal arches 1 and 2 (outlined). (J, K) Quantification of pHH3+ labeled cells illustrating no global overall decrease in mutants embryos compared to controls, but a significant decrease in the percentage of pHH3+ cells in pharyngeal arches 1 and 2 in *polr1c* mutant embryos. *polr1d* mutant embryos showed a similar level of proliferation as *polr1c* mutants. Scale bar = 100 μm. * = p < 0.01 and error bars represent 95% confidence intervals.
(TIF)

S6 Fig *tp53* inhibition reduces cell death in 24 hpf *polr1c* mutant embryos. (A-D) TUNEL staining in *polr1c; tp53* embryos reveals a decrease in cell death depending on the dosage of *tp53*. (E-F) Cross sections confirm diminished levels of cell death within and around the neural tube. Scale bars = 100 μm.
(TIF)

S7 Fig *tp53* inhibition reduces cell death in 24 hpf *polr1d* mutant embryos. (A-D) TUNEL staining in *polr1d; tp53* embryos reveals a decrease in cell death depending on the dosage of *tp53*. (E-F) Cross sections confirm diminished levels of cell death around the neural tube. Scale bars = 100 μm.
(TIF)

S8 Fig *tp53* inhibition improves the phenotype of *polr1c* mutant embryos. (A-I) Alcian blue and alizarin red staining reveal improved cartilage formation and patterning in *polr1c*^-/-^; *tp53*^-/-^ embryos compared to *polr1c*^-/-^; *tp53*^+/+^ embryos. Abbreviations: M, Meckel’s cartilage; pq, palatoquadrate; ch, ceratohyal; cb, ceratobranchial. Scale bars = 200 μm
(TIF)

S9 Fig *tp53* inhibition results in a dosage dependent rescue of the *polr1c* and *polr1d* mutant phenotypes. The percentage of embryos with wild-type (wt, orange), mild (green), and severe (blue) phenotypes upon *tp53* inhibition are shown. The percentage of embryos with wild-type appearance upon removal of one copy of *tp53* is around 20% in *polr1c* and *polr1d* embryos. This percentage increases with removal of both copies of *tp53*, with the percentage of severe phenotype accounting for less than 10% of the mutants.
(TIF)
