# Supplementary figures and images for: The Roles of RNA Polymerase I and III Subunits Polr1c and Polr1d in Craniofacial Development and in Zebrafish Models of Treacher Collins Syndrome
Source: PLoS Genet. 2016 Jul 22;12(7):e1006187. doi: 10.1371/journal.pgen.1006187 (PMC4957770; doi:10.1371/journal.pgen.1006187)

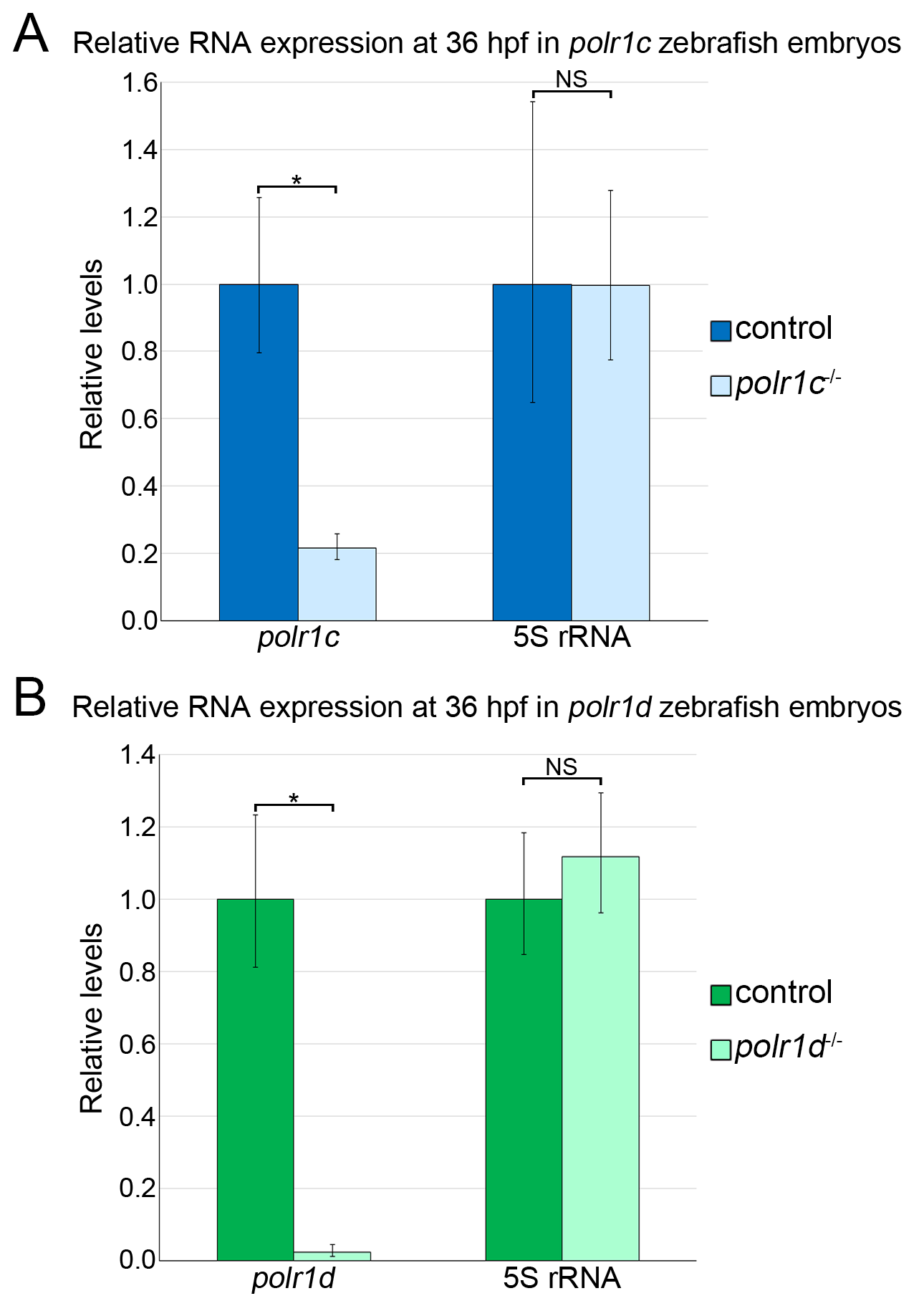

Supplement: S1 Fig — (A) polr1c reduced by approximately 80% in homozygous mutant embryos while levels of 5S rRNA remain unchanged. (B) polr1d is reduced by approximately 97% in homozygous mutant embryos while 5S rRNA levels are unchanged. * = p < 0.05 and error bars represent 95% confidence intervals. (TIF) [file pgen.1006187.s001.tif]

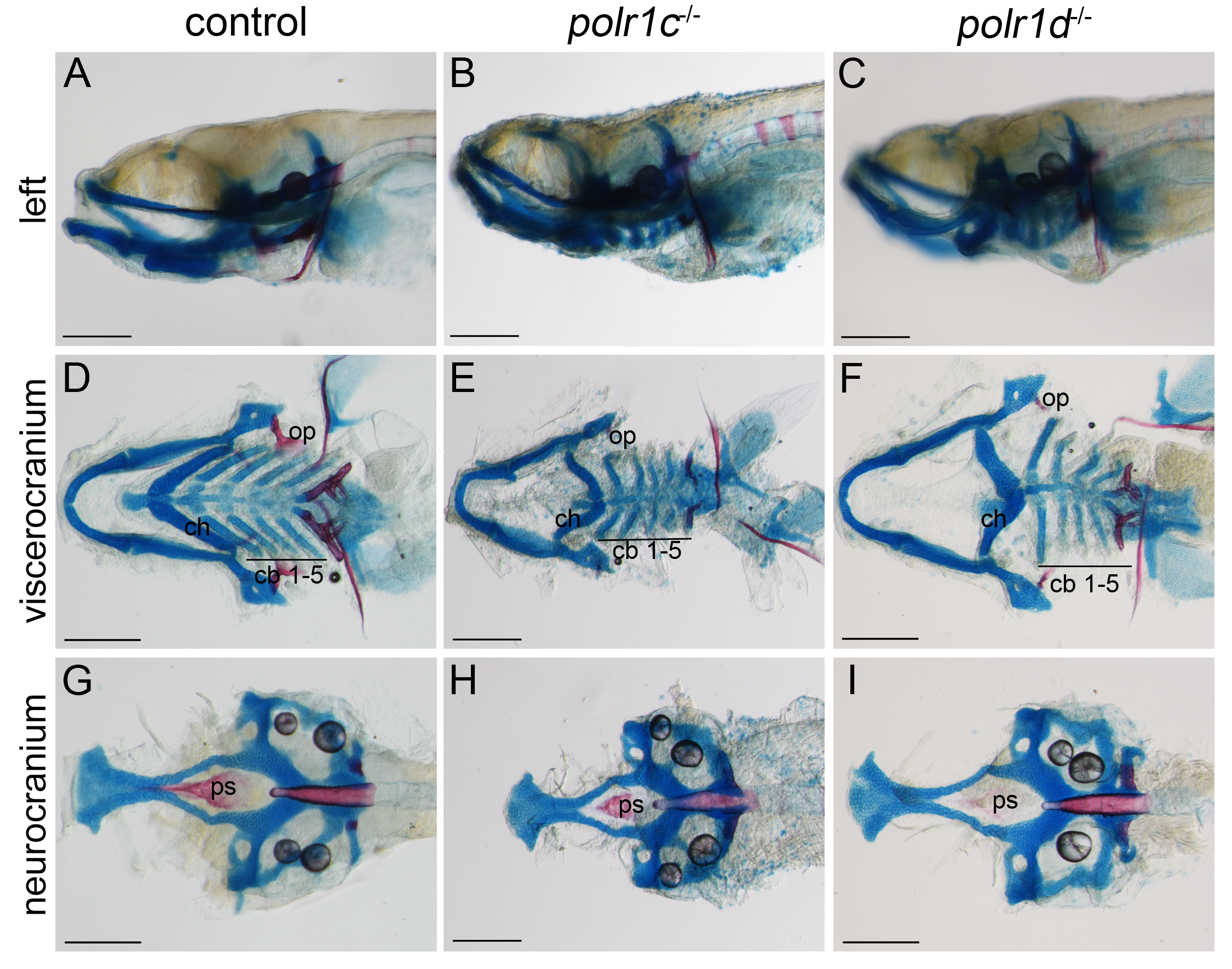

Supplement: S2 Fig — (A-C) Alcian blue and Alizarin red staining reveals diminished cartilage and bone formation in polr1c and polr1d mutant embryos. (D-F) Dissection of the viscerocranium revealed mispatterning of the ceratohyal (ch), and hypoplasia of the ceratobranchial cartilages (cb) in polr1c and polr1d mutants. There is also hypoplasia of bone elements including the opercles (op) and pharyngeal teeth. (G-H) Dissection of the neurocranium reveals reduced ossification of the parasphenoid (ps) in mutant embryos. Scale bar = 200 μm. (TIF) [file pgen.1006187.s002.tif]

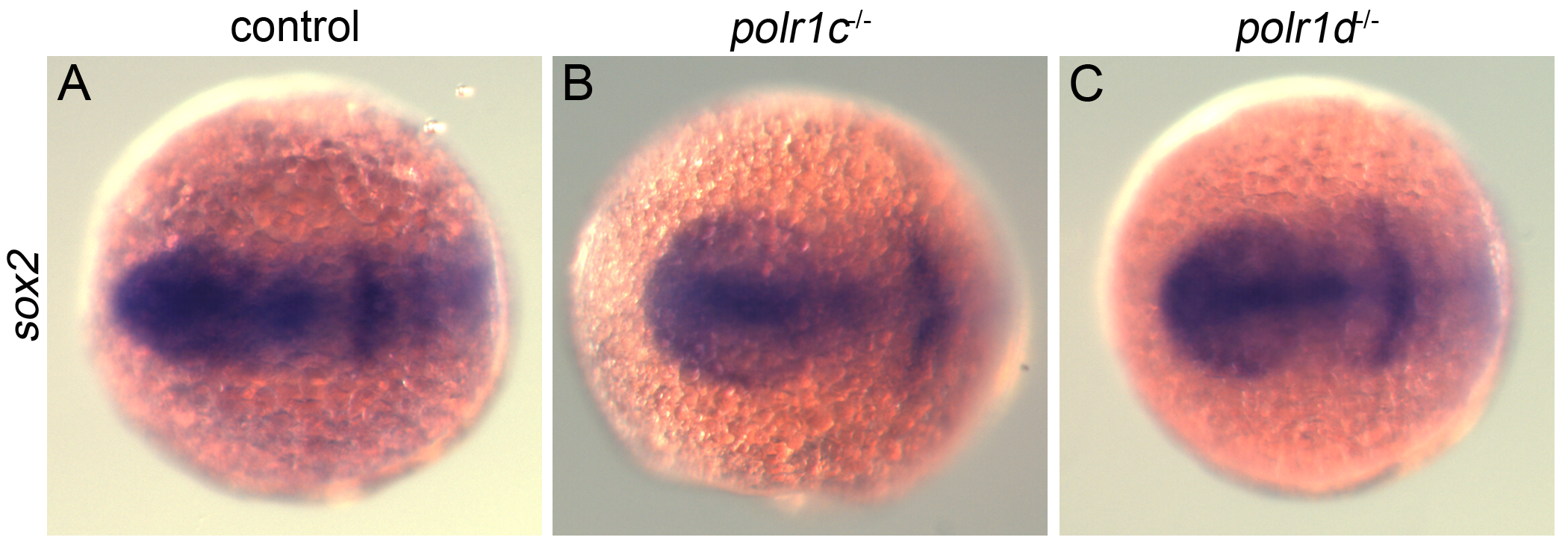

Supplement: S3 Fig — (A-C) polr1c-/- and polr1d-/- mutant embryos show similar expression of sox2 compared to controls as revealed by in situ hybridization. This indicates that neural plate formation, a precursor to formation of neural crest cells, occurs in mutant embryos. (TIF) [file pgen.1006187.s003.tif]

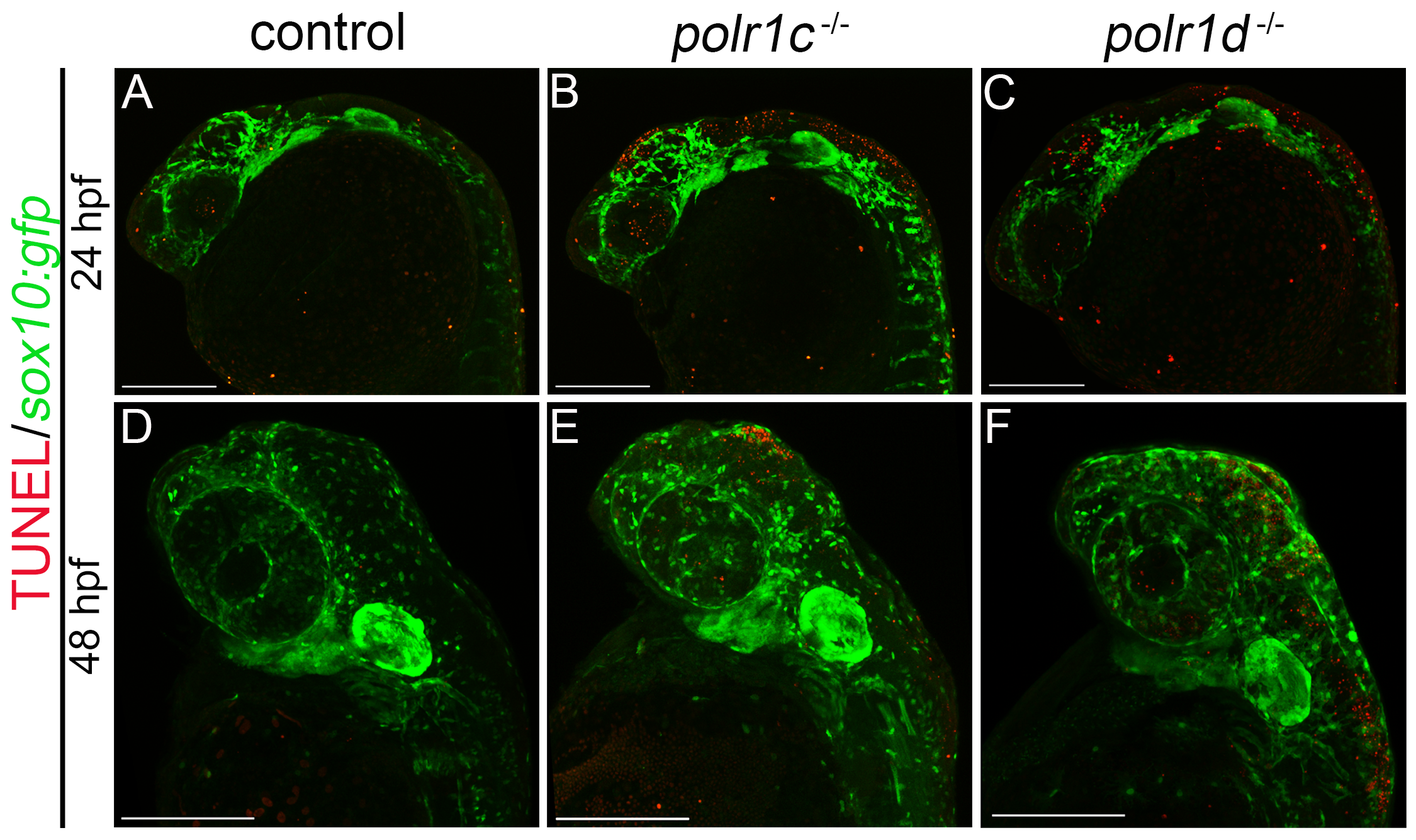

Supplement: S4 Fig — (A-D) TUNEL staining in sox10:gfp embryos shows increased cell death in mutant embryos which does not co-localize with the migratory NCC population. At 24 hpf, increased cell death can be seen in the neuroepithelial region of polr1c-/- and polr1d-/- embryos. (E-F) At 48 hpf, increased cell death can be observed in regions of the brain and eye of mutant embryos, but not within the pharyngeal arches. Scale bar = 200 μm. (TIF) [file pgen.1006187.s004.tif]

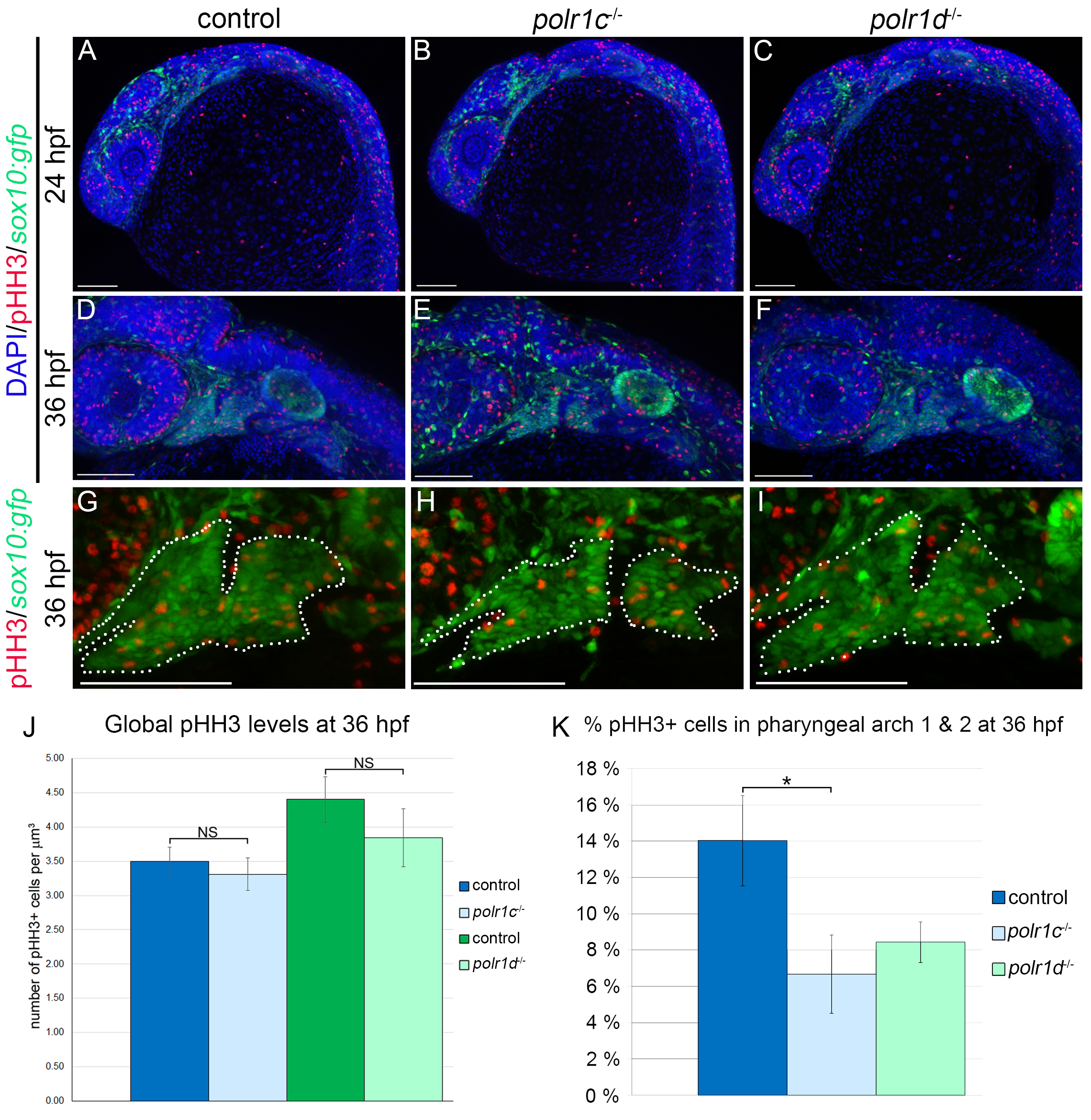

Supplement: S5 Fig — (A-C) similar levels of pHH3 staining are present in control, polr1c-/- and polr1d-/- embryos at 24 hpf. (D-F) Proliferation at 36 hpf also occurs globally at broadly similar levels in controls and mutant embryos, but there differences in the number of pHH3+ cells within pharyngeal arches 1 and 2. (G-I) Magnified views of pharyngeal arches 1 and 2 (outlined). (J, K) Quantification of pHH3+ labeled cells illustrating no global overall decrease in mutants embryos compared to controls, but a significant decrease in the percentage of pHH3+ cells in pharyngeal arches 1 and 2 in polr1c mutant embryos. polr1d mutant embryos showed a similar level of proliferation as polr1c mutants. Scale bar = 100 μm. * = p < 0.01 and error bars represent 95% confidence intervals. (TIF) [file pgen.1006187.s005.tif]

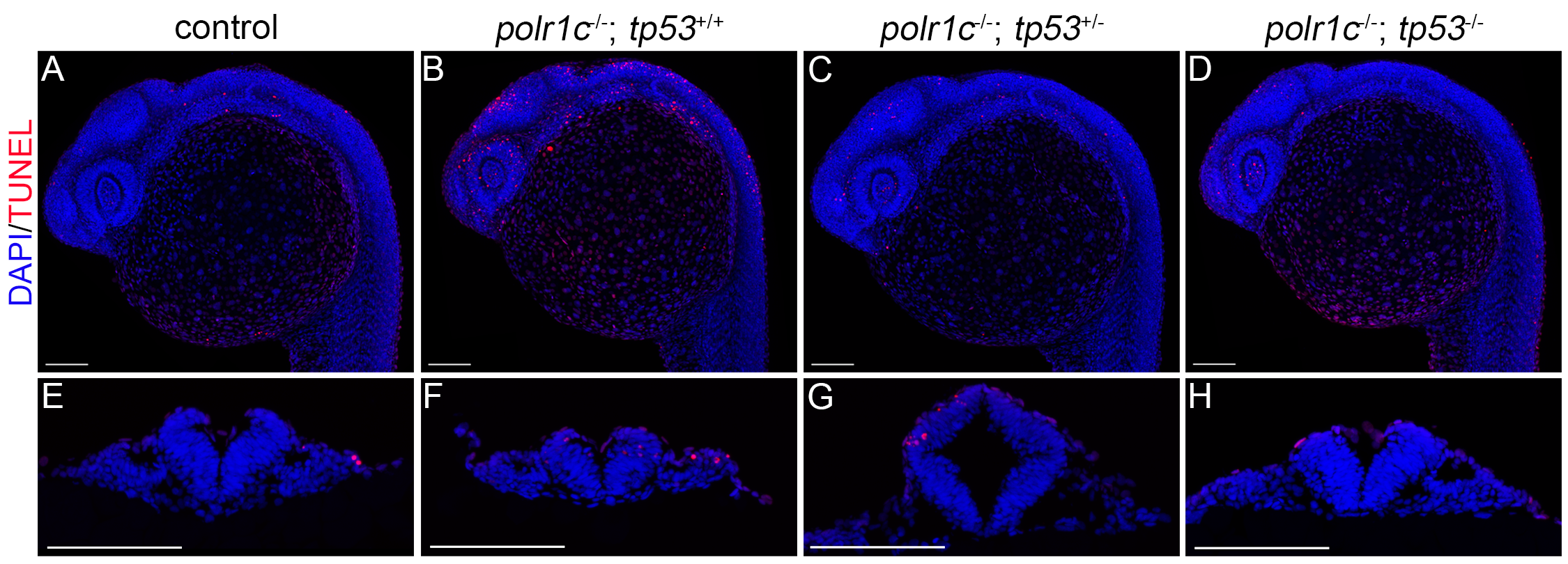

Supplement: S6 Fig — (A-D) TUNEL staining in polr1c; tp53 embryos reveals a decrease in cell death depending on the dosage of tp53. (E-F) Cross sections confirm diminished levels of cell death within and around the neural tube. Scale bars = 100 μm. (TIF) [file pgen.1006187.s006.tif]

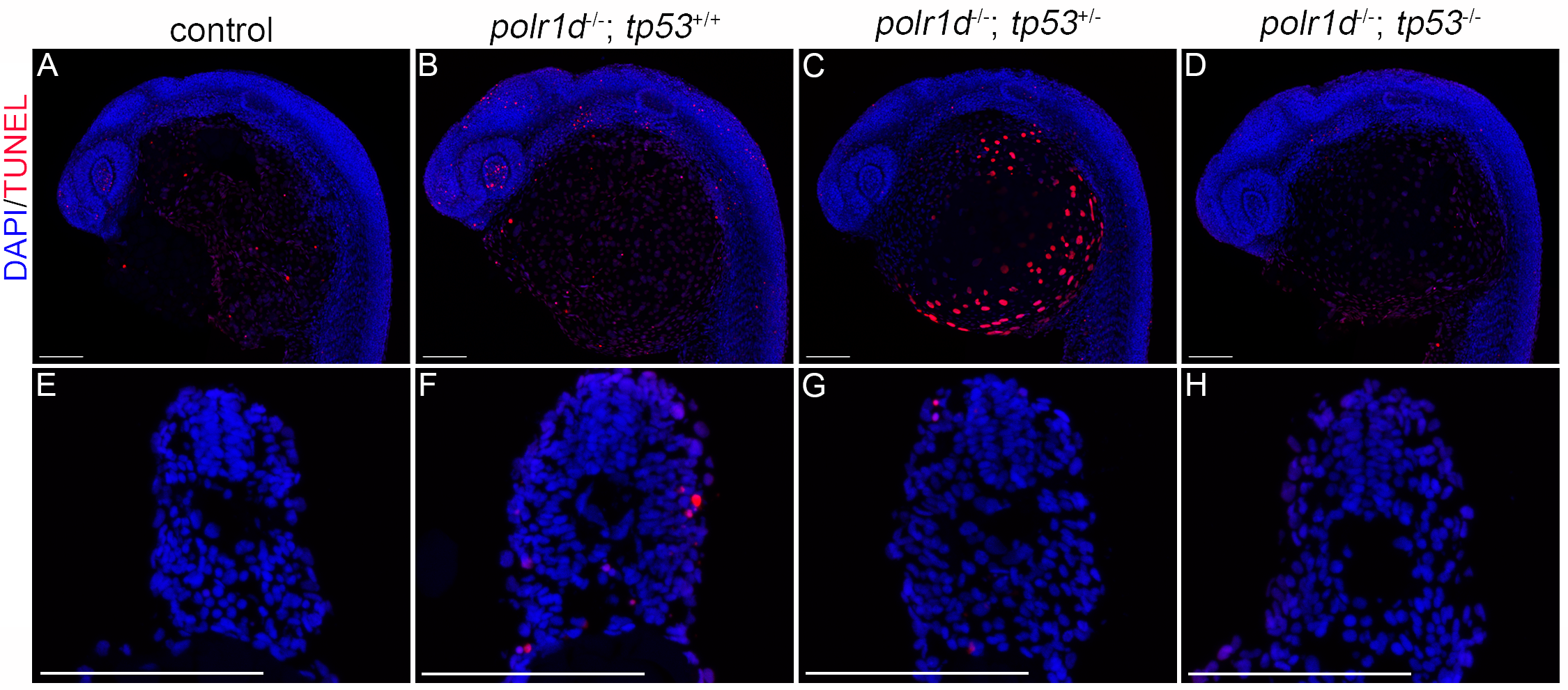

Supplement: S7 Fig — (A-D) TUNEL staining in polr1d; tp53 embryos reveals a decrease in cell death depending on the dosage of tp53. (E-F) Cross sections confirm diminished levels of cell death around the neural tube. Scale bars = 100 μm. (TIF) [file pgen.1006187.s007.tif]

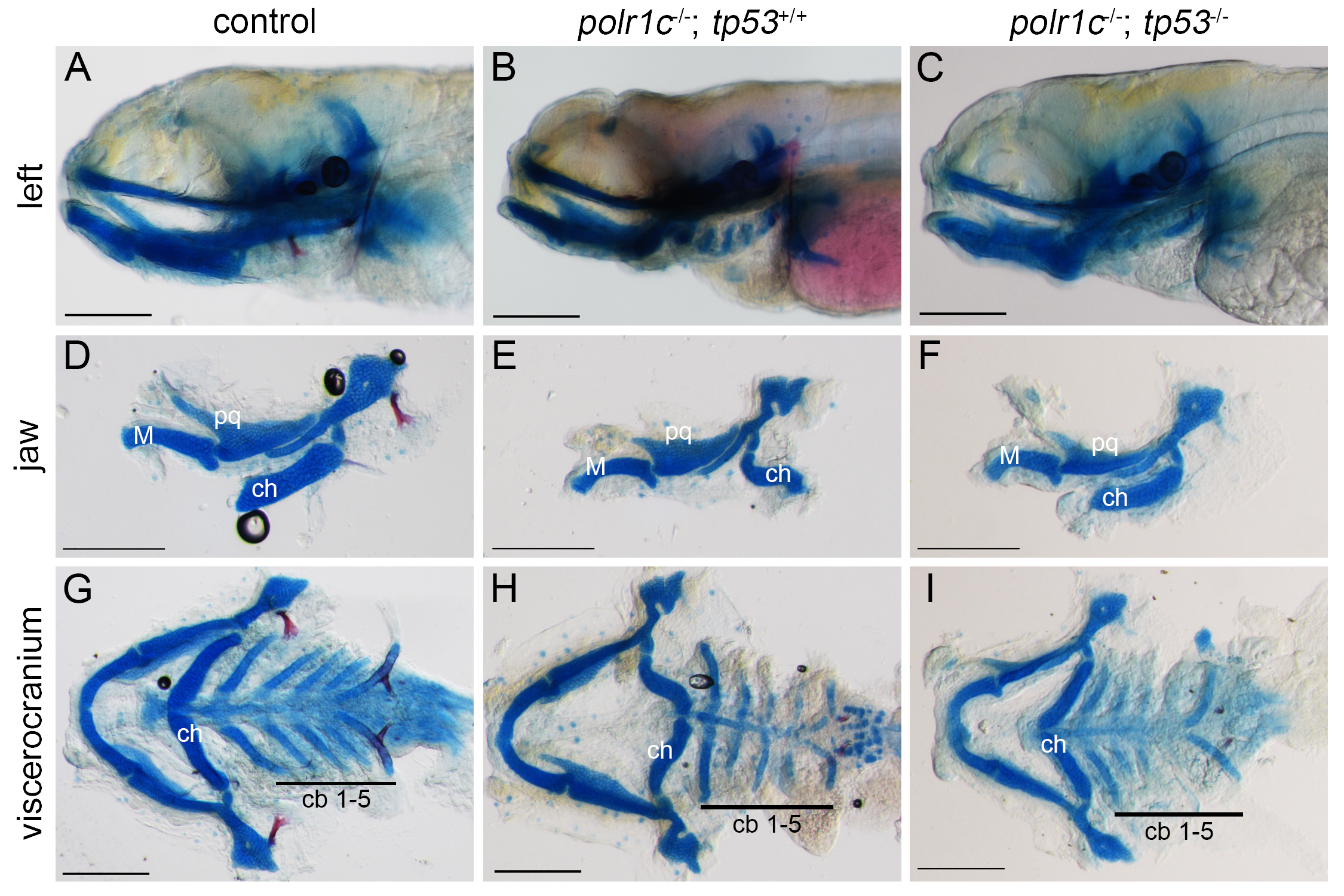

Supplement: S8 Fig — (A-I) Alcian blue and alizarin red staining reveal improved cartilage formation and patterning in polr1c-/-; tp53-/- embryos compared to polr1c-/-; tp53+/+ embryos. Abbreviations: M, Meckel’s cartilage; pq, palatoquadrate; ch, ceratohyal; cb, ceratobranchial. Scale bars = 200 μm (TIF) [file pgen.1006187.s008.tif]

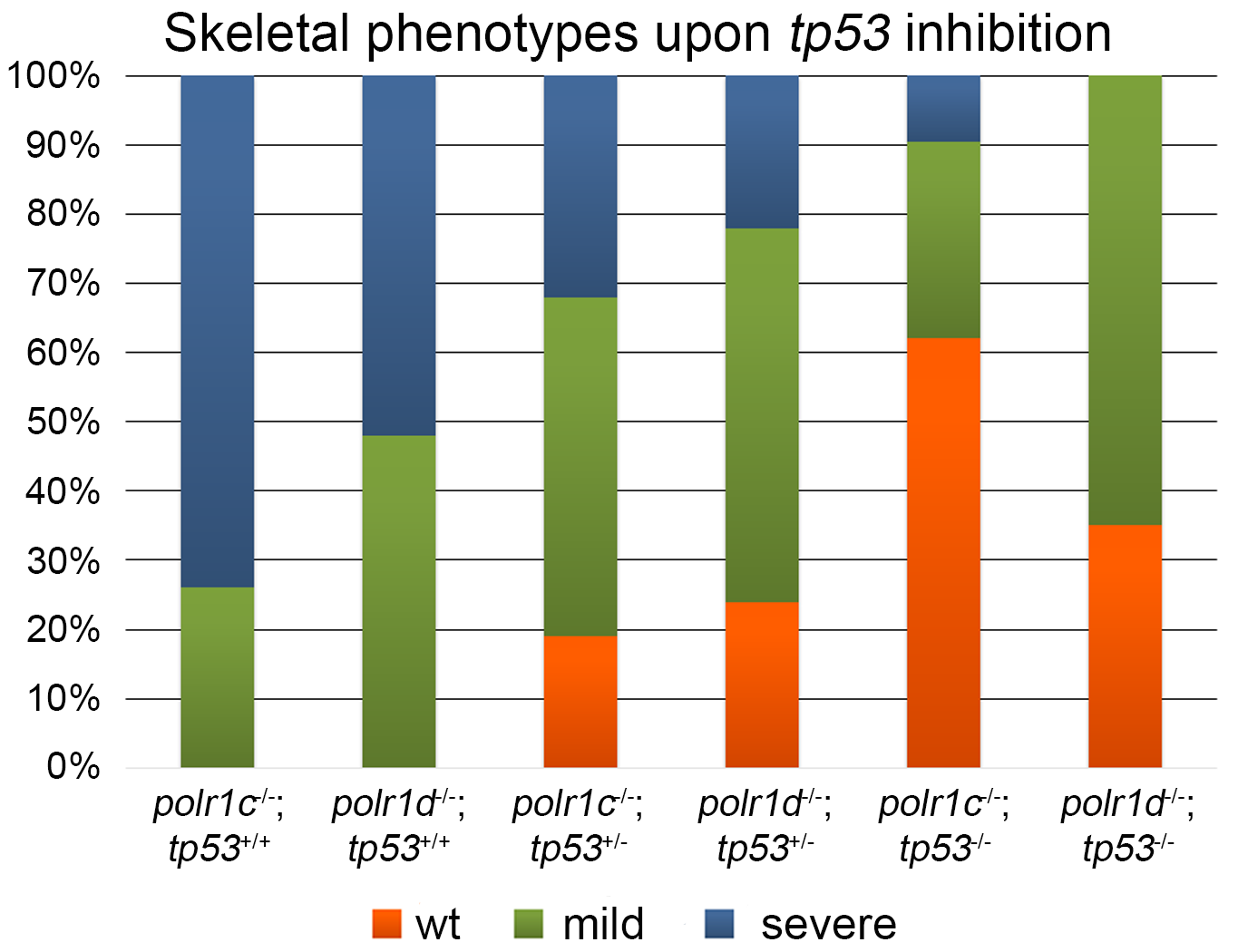

Supplement: S9 Fig — The percentage of embryos with wild-type (wt, orange), mild (green), and severe (blue) phenotypes upon tp53 inhibition are shown. The percentage of embryos with wild-type appearance upon removal of one copy of tp53 is around 20% in polr1c and polr1d embryos. This percentage increases with removal of both copies of tp53, with the percentage of severe phenotype accounting for less than 10% of the mutants. (TIF) [file pgen.1006187.s009.tif]
